# Copper ions inhibit pentose phosphate pathway function in *Staphylococcus aureus*

**DOI:** 10.1371/journal.ppat.1011393

**Published:** 2023-05-26

**Authors:** Javiera Norambuena, Hassan Al-Tameemi, Hannah Bovermann, Jisun Kim, William N. Beavers, Eric P. Skaar, Dane Parker, Jeffrey M. Boyd

**Affiliations:** 1 Department of Biochemistry and Microbiology, Rutgers University, New Brunswick, New Jersey, United States of America; 2 Department of Pathology, Immunology and Laboratory Medicine, Center for Immunity and Inflammation, Rutgers New Jersey Medical School, Newark, New Jersey, United States of America; 3 Department of Pathology, Microbiology, and Immunology, Vanderbilt University Medical Center, Nashville, Tennessee, United States of America; University of Tubingen, GERMANY

## Abstract

To gain a better insight of how Copper (Cu) ions toxify cells, metabolomic analyses were performed in *S*. *aureus* strains that lacks the described Cu ion detoxification systems (Δ*copBL* Δ*copAZ*; *cop*^-^). Exposure of the *cop*^-^ strain to Cu(II) resulted in an increase in the concentrations of metabolites utilized to synthesize phosphoribosyl diphosphate (PRPP). PRPP is created using the enzyme phosphoribosylpyrophosphate synthetase (Prs) which catalyzes the interconversion of ATP and ribose 5-phosphate to PRPP and AMP. Supplementing growth medium with metabolites requiring PRPP for synthesis improved growth in the presence of Cu(II). A suppressor screen revealed that a strain with a lesion in the gene coding adenine phosphoribosyltransferase (*apt*) was more resistant to Cu. Apt catalyzes the conversion of adenine with PRPP to AMP. The *apt* mutant had an increased pool of adenine suggesting that the PRPP pool was being redirected. Over-production of *apt*, or alternate enzymes that utilize PRPP, increased sensitivity to Cu(II). Increasing or decreasing expression of *prs* resulted in decreased and increased sensitivity to growth in the presence of Cu(II), respectively. We demonstrate that Prs is inhibited by Cu ions *in vivo* and *in vitro* and that treatment of cells with Cu(II) results in decreased PRPP levels. Lastly, we establish that *S*. *aureus* that lacks the ability to remove Cu ions from the cytosol is defective in colonizing the airway in a murine model of acute pneumonia, as well as the skin. The data presented are consistent with a model wherein Cu ions inhibits pentose phosphate pathway function and are used by the immune system to prevent *S*. *aureus* infections.

## Author summary

Antimicrobial resistance is a global health emergency. There is an increasing demand to develop new drugs and repurpose chemotherapeutics to prevent and treat infections. Metals and metal ions have been used as effective antimicrobials for millennia. This effectiveness is likely due to their ability to simultaneously inhibit multiple cellular processes. Copper (Cu) is a metal that has been used by humans as a prophylactic. Furthermore, the human immune system utilizes Cu to aid bacterial killing or growth inhibition. The human pathogen *Staphylococcus aureus* has two described Cu ion detoxification systems, underscoring the efficacy of Cu ions to negatively impact *S*. *aureus* growth and survival. Questions remain about how bacteria are toxified by Cu ions. Understanding how Cu kills and inhibits bacterial growth will provide useful information for designing and implementing antimicrobial chemotherapy to decrease bacteria burden. Herein, we demonstrate that the essential enzyme Prs is a target of Cu ions in *S*. *aureus*. Using mouse models of pneumonia and skin infections, we demonstrate that *S*. *aureus* that cannot effectively remove Cu ions from the inside of the cell are defective in colonizing tissues.

## Introduction

Copper (Cu), in both its ionic and metallic forms, has been used as an antimicrobial for millennia. Several studies have demonstrated that bacterial strains that are genetically modified to have a decreased ability to remove Cu ions from their cytosols have decreased survival on copper or copper alloy surfaces [1–3]. A Cu ion probe was used to demonstrate that bacteria aid the release Cu ions from dry or moist metallic Cu metal surfaces which then accumulate in their cytosol [4]. These studies suggest that when bacteria contact metallic Cu surfaces, Cu ions are liberated, are mobilized to the cytosol, and contribute to killing.

Cu ions have significant roles in biology including acting as an enzymatic cofactor for redox reactions; however, an excess of intracellular Cu ions cause intoxication [5]. It has been shown that intracellular Cu ions can cause demetallation and/or mismetalation of proteins with solvent exposed metal-binding sites [6, 7]. Cu ions can disrupt iron-sulfur (FeS) clusters [6] resulting in Fe release which can potentiate oxidative stress [8]. Cu can also inhibit the maturation of FeS proteins by binding to synthesis and assembly factors [9–12]. Cu(I) and Fe(II) can catalyze the production of hydroxyl radicals resulting in oxidative stress which can damage biological polymers [13, 14]. Although, *in vivo*, cytosolic Cu ion accumulation does not appear to cause DNA damage, suggesting that ROS production is not a primary mechanism for Cu ion induced killing or growth inhibition [15].

Cu ions aid humans in preventing and clearing infections [16–18]. Cu ions accumulate at sites of inflammation [17], within macrophage intracellular vesicles [19], and in phagosomes [20] where they aid bacterial clearance [21]. *Staphylococcus aureus* strains that have been genetically modified to be defective in exporting Cu from the cytosol have decreased survival in macrophages and whole blood [22, 23]. Proper Cu ion removal from the *S. aureus* cytosol was required for complete fitness in a murine model of urinary tract of infection [24].

To aid in the prevention of cytosolic Cu ion accumulation, *S*. *aureus* USA300 possess two Cu-exporting ATPases coded by *copA* and *copB*. CopA is a P1-type ATPase [25] and it is in an operon with *copZ*. CopZ binds Cu(I) and delivers it to CopA for export [26]. CopB is also a P1-type ATPase and it is in an operon with *copL* [5, 23]. CopL is an extracellular Cu ion binding lipoprotein that likely prevents the entry of Cu to the cytoplasm [5]. The *copBL* operon is coded within the ACME transposable element found in some *S. aureus* strains [27].

The mechanisms by which cytosolic Cu ion accumulation intoxicates bacterial cells appears to be multifaceted and it is not entirely clear which processes are inhibited resulting in cell death [10, 28–30]. For this study, we used a strain which lacks the described Cu detoxification systems (Δ*copBL* and Δ*copAZ*; strain named *cop*^-^) to analyze the effect of cytosolic Cu ion accumulation and to identify metabolic bottlenecks to predict enzymes or pathways that are inhibited [31]. Using unbiased approaches, we demonstrate that an inability to remove Cu ions from the cytosol results in defective function of the pentose phosphate pathway (PPP). Genetic data suggest that altering the pool of phosphoribosyl pyrophosphate (PRPP) modulates the sensitivity of *S*. *aureus* to growth in the presence of Cu(II). We also establish that Cu(I) poisons the enzyme phosphribosylpyrophosphate synthetase (Prs), which produces the essential metabolite PRPP. Finally, we corroborated the importance of cytosolic Cu-detoxification systems in colonization of skin and airways using murine models of infection.

## Results

### Cu(II) treatment perturbs carbon flow through central metabolic pathways

To better understand the effects of Cu(II) treatment on intracellular metabolite pools we performed unbiased metabolomic analyses on the *S*. *aureus cop*^-^ strain. We exposed an exponentially growing culture to 5 μM Cu(II) for 30, 60, or 180 minutes while growing in tryptic soy broth (TSB). We picked this concentration of Cu(II) because it did not significantly affect the growth of the *cop*^-^ strain and we previously demonstrated that this amount of Cu(II) was sufficient to cause cytosolic accumulation in the *cop*^-^ strain compared to the WT [32]. Although the MIC for Cu(II) (2 mM) is quite high for the *cop*^-^ strain cultured in TSB, incubating with a concentration of Cu(II) significantly lower than 2 mM still retards growth. We utilized 5 μM Cu(II) to ensure that any significant differences in metabolite concentration were the result of Cu ions and not a slow growth phenotype.

**Fig 1:**
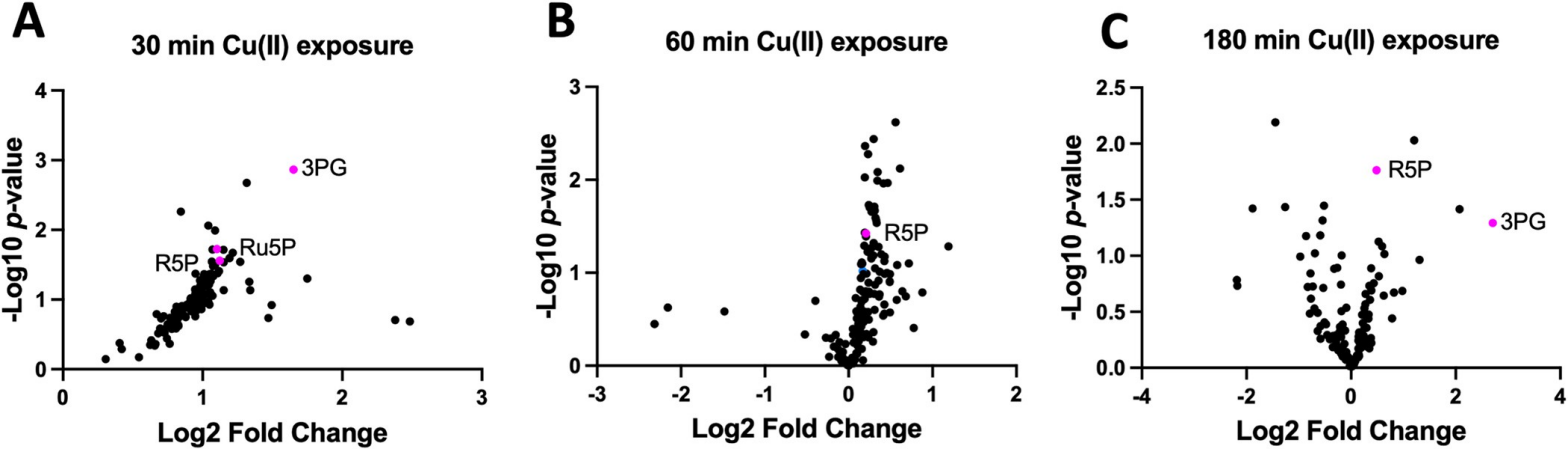
Growth with Cu(II) alters metabolite pools. Volcano plots of Log_2_ fold change of metabolite abundances isolated from *cop*^-^ cells after 30 (panel A), 60 (panel B), and 180 (panel C) minutes after the addition of Cu(II) to the growth media. Data represent the average of three biological replicates.

Metabolites that were significantly altered when compared to cultures treated with the vehicle control at each time point are listed in Table 1 and illustrated in Fig 1. The complete list of metabolite concentrations can be found in S1 Table. The number of significantly altered metabolites was largest after 30 minutes of exposure and then decreased over time. These data suggest that the metabolic network of *S*. *aureus* is responsive to growth in the presence of Cu(II) and adapts to redirect metabolic flux to help buffer against the effects of Cu ions.

**Table 1:** Select metabolites that are significantly altered in the *cop*^-^ strain when cultured in presence of 5 μM Cu(II)*.

30-minute exposure		60-minute exposure		180-minute exposure	
metabolite	Log2 fold-change	metabolite	Log2 fold-change	metabolite	Log2 fold-change
Aconitate	1.1	AICA-riboside	0.3	Adenine	1.2
ADP	1.1	Alanine	0.2	Dihydroxyacetone phosphate	2.1
Arginine	1.0	Cystathionine	0.2	3-Phosphoglycerate	2.7
Dihydroxyacetone phosphate	1.1	Glutamine	0.5	Ribose 5-phosphate	0.5
Fructose-1 6-bisphosphate	1.8	Isoleucine	0.4	Serine	-0.5
Fructose-6-phosphate	1.2	Leucine	0.3	Threonine	-0.5
Glucose	1.3	Methionine	0.3		
Glucose 1-phosphate	1.1	Mevalonate	0.3		
Glucose 6-phosphate	1.1	Pantothenate	0.4		
Glycerate	1.2	Ribose 5-phosphate	0.2		
Isocitrate	1.0	Serine	0.6		
Lysine	1.0	Threonine	0.6		
NADH	1.0	Valine	0.3		
NADP^+^	1.1				
Orotate	1.2				
3-Phosphoglycerate	1.7				
Pyruvate	1.1				
Ribitol	1.1				
Ribose 5-phosphate	1.1				
Ribulose-5-phosphate	1.1				
Threonine	1.1				
UDP-Glucose	1.2				
3-Ureidopropionic acid	1.3				

*Experiment was conducted using biological triplicates. All metabolites listed were significantly (students two tailed t-test p < 0.051) altered compared to the untreated sample. A full list of metabolites can be found in S1 Table

**Fig 2:**
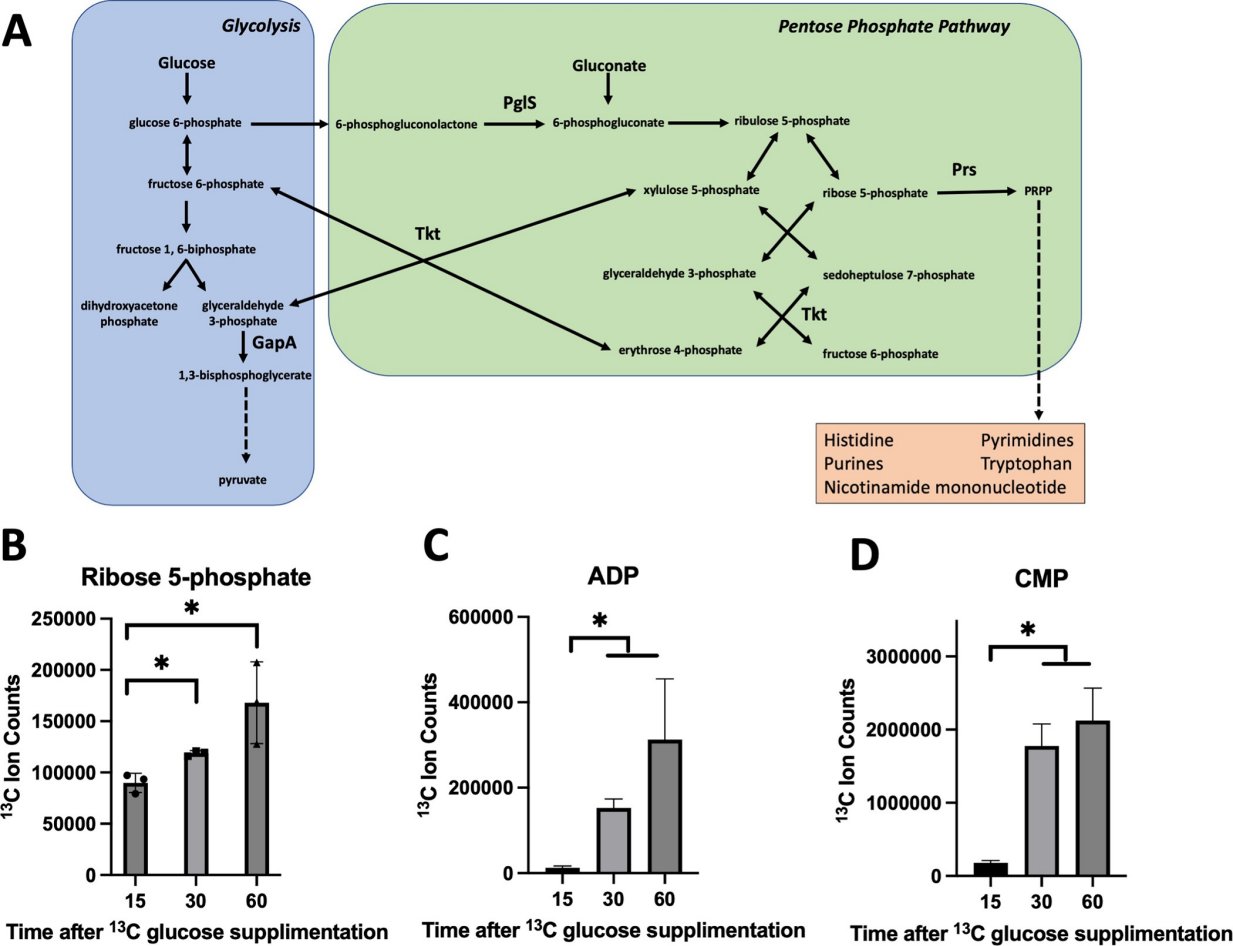
Carbon from glucose is fluxed through the pentose phosphate pathway when growing in TSB medium. Panel A, schematic of the interplay between the glycolytic and pentose phosphate pathways. Key enzymes and metabolites are highlighted. Panels B, C, and D, concentrations of total ^13^C carbon atoms in select metabolites after growth in TSB medium containing ^13^C glucose. Data represent the average of three biological replicates and standard deviations are displayed. Student’s t-tests were performed on the data and * indicates p < 0.05.

After 30 minutes of growth in the presence of Cu(II) there was a significant accumulation of the glycolytic metabolites glucose, glucose 6-phosphate, dihydroxyacetone phosphate, 3-phosphoglycerate (3PG) and fructose 6-phosphate. These metabolites are processed upstream of glyceraldehyde 3-phosphate dehydrogenase (GapA) which was previously shown to be inhibited by Cu ions *in vivo* [33]. These metabolites would also be expected to accumulate if there was a Cu-induced bottleneck in the PPP (Fig 2A). Consistent with a block in the PPP, there was an increase in the accumulation of ribose 5-phosphate (R5P) and ribulose 5-phosphate (Ru5P) which are key metabolites of the pentose phosphate pathway. The only metabolite that accumulated in all three time points was R5P. Ru5P, serine, 3-phosphoglycerate, and dihydroxyacetone phosphate accumulated in two of the time points. These data are consistent with the hypothesis that Cu ion accumulation alters carbon flux through glycolysis and/or the PPP.

### Carbon from glucose is fluxed through the pentose phosphate pathway

We tested the hypothesis that carbon from glucose is fluxed through the PPP to metabolites requiring R5P synthesis when *S*. *aureus* is grown on TSB medium. We cultured cells in a label-free TSB medium to exponential growth phase and transferred cells to ^13^C_6_-glucose TSB. Samples were taken at 15, 30 and 60 minutes after the switch and metabolites were quantified. After fifteen minutes ^13^C was incorporated into metabolites that are intermediates in glycolysis and the PPP pathways (Fig 2 and S2 Table). This list of metabolites included R5P. There was also an increase in ^13^C in metabolites derived from R5P including purines and pyrimidines (Table 2). For many of the metabolites, the concentration of incorporated ^13^C atoms increased as a function of time (Fig 2). Likewise, the total amount of ^13^C atoms in the nicotinamide dinucleotides NADH and NAD^+^ increased over-time (S1 Fig). In addition, the number of ^13^C atoms per individual NADH and NAD^+^ molecule increased over time. We were not able to determine which atoms were labeled with ^13^C in the NAD^+^ or NADH (or any other molecule), but we did witness the formation of molecules that contained label in 12, 13, or 14 of the 21 total carbon atoms (five carbons from adenine, 6 from nicotinamide, 10 from ribose) suggesting that the ribose component was labeled with ^13^C from glucose.

**Table 2:** Select metabolites containing significant quantities of ^13^C after growth with ^13^C glucose*.

Glycolysis	Purines	Pyrimidines
glucose 6-phosphate	GMP	CMP
3-phosphoglycerate	dGMP	UMP
glycerol 3-phosphate	AMP	dUMP
phosphoenolpyruvate	dAMP	dTMP
pyruvate	AMP	uracil
	AICAR	
Pentose phosphate pathway	AICA-riboside	
Ribose 5-phosphate		Nicotinamide cofactors
sedoheptulose 7-phosphate		NAD^+^
Ribose		NADH

*a full list of metabolites can be found in S2 Table.

### Requiring carbon to be fluxed though the PPP increases sensitivity to Cu(II)

**Fig 3:**
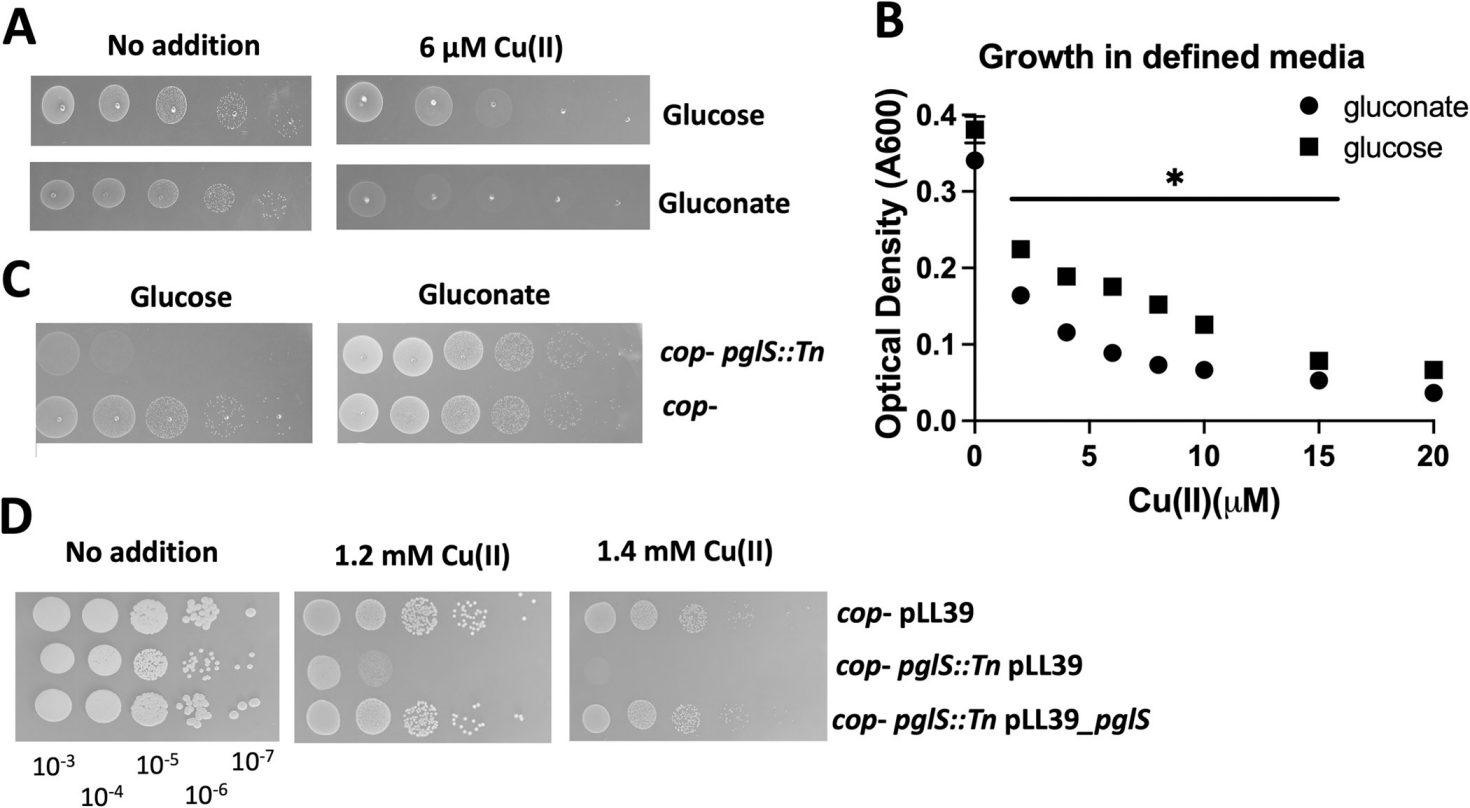
Growth with Cu(II) alters pentose phosphate pathway function. Panel A, the *cop*^-^ strain was serial diluted and spot plated on chemically defined media containing either glucose or gluconate as the primary carbon source with and without 6 μM Cu(II). Panel B, the optical densities of cultures of the *cop*^-^ strain after 18 hours of growth in defined liquid media with 11 mM glucose or gluconate as the primary carbon source and 0–20 μM Cu(II) are presented. The data presented represent the average of three biological replicates and standard deviations are displayed; however, they are too small to be seen for most data points. Student’s t-tests were performed between culture optical density readings for glucose and gluconate growth at each individual Cu(II) concentration and * indicates p < 0.05. A significant difference in final optical density was noted for all Cu(II) concentrations except for 0 and 20 μM Cu(II). Panel C, the *cop*^-^ and *cop*^-^ *pgl*::*Tn* strains were serial diluted and spot plated on defined media containing either 11 mM glucose or gluconate as the primary carbon source. Panel D, the *cop*^-^ and *cop*^-^ *pgl*::*Tn* strains containing either pLL39 or pLL39_*pglS* were serial diluted and spot plated on TSA media containing 0 or 1.2 mM Cu(II). Photos of representative experiments displayed.

Either glucose or gluconate can be used to produce R5P. Glucose can be processed through glycolysis or the PPP; however, gluconate can only be processed through the PPP in *S*. *aureus* (Fig 2A). We hypothesized that if growth with Cu(II) results in decreased PPP function, *S*. *aureus* would have decreased growth when the primary carbon source was gluconate as compared to glucose. We spot plated the *cop*^-^ strain in the presence and absence of Cu(II) on defined media containing either glucose or gluconate as a primary carbon source (Fig 3A). Growth was similar if glucose or gluconate were primary carbon sources in the absence of exogenously supplied Cu(II); however, there was a more profound growth defect on gluconate medium when the media were supplemented with Cu(II). A similar trend was noted when cells were cultured in liquid defined media with either glucose or gluconate as primary carbon sources and various concentrations of Cu(II) (Fig 3B).

We next tested the hypothesis that decreasing flux from glucose into the PPP would increase the sensitivity of *S*. *aureus* to growth in the presence of Cu(II) when glucose was the primary carbon source. To this end, we created a *cop*^*-*^ strain with a lesion in the gene that encodes for 6-phosphogluconolactonase (*pglS*; SAUSA300_1902), which hydrolyzes 6-phosphogluconolactone to 6-P gluconate (Fig 2A). Growth on gluconate as the primary carbon source should bypass the need for PglS, whereas growth with glucose partially relies on PglS to flux carbon from glucose into the PPP. It was previously demonstrated that conversion of 6-phosphogluconolactone to 6-P gluconate can occur spontaneously, making *pglS* non-essential for growth with glucose [34]. We cultured the *cop*^*-*^ *pglS*::*Tn* and *cop*^*-*^ strains on defined media with either glucose or gluconate as carbon source. The *cop*^*-*^ *pglS*::*Tn* mutant grew similar to the *cop*^*-*^ strain in defined medium with gluconate as a carbon source, but had a slow growth phenotype when glucose was provided as a carbon source (Fig 3C) supporting a role for PglS in PPP. Glucose is the primary carbon source is TSB. The *cop*^*-*^ and *cop*^*-*^ *pglS*::*Tn* strains behaved similar when cultured on tryptic soy agar (TSA) medium; however, supplementation of the TSA medium with Cu(II) resulted in a significant growth defect in the *cop*^*-*^ *pglS*::*Tn* strain when compared to the *cop*^*-*^ strain and the phenotype could be genetically complemented (Figs 3D and S2).

We further analyzed the necessity of a proper functioning PPP for Cu resistance using a strain lacking transketolase (Tkt). The *cop*^*-*^ Δ*tkt*::*kan* mutant strain had a small colony phenotype on TSA medium making it difficult to accurately compare the growth with that of the *cop*^*-*^ strain when cultured with Cu(II). Therefore, we determined the minimal inhibitory concentration (MIC) for Cu(II) when growing in TSB media. Whereas the MIC for Cu(II) was 2 mM for the *cop*^*-*^ strain, it was 1 mM for the *cop- pglS*::*Tn* and *cop- Δtkt*::*kan* strains. Taken together, these data suggest that perturbing carbon flux though the PPP alters the sensitivity of *S*. *aureus* to Cu(II).

### Suppressor analysis suggests that sparing PRPP protects against Cu(II) intoxication

We generated a transposon mutant library in the *cop*^*-*^ background. The library was plated on TSA medium containing 2.5 mM Cu(II) which is a non-permissive growth concentration of Cu(II) for the *cop*^*-*^ strain. Transposon insertions that led to suppression of the Cu(II) sensitivity phenotype of the *cop*^*-*^ strain were mapped to three genes: *mntA*, *ispA*, and *apt*. We previously described a role for MntABC in Cu(II) resistance [31]. The two *apt*::*Tn* insertions were located at the TA/AT site located at +438 (*apt*::*Tn*). The *apt* gene is downstream of *recJ*, in an apparent operon. The role of *ispA* in Cu ion homeostasis is unknown and not the focus of this study.

**Fig 4:**
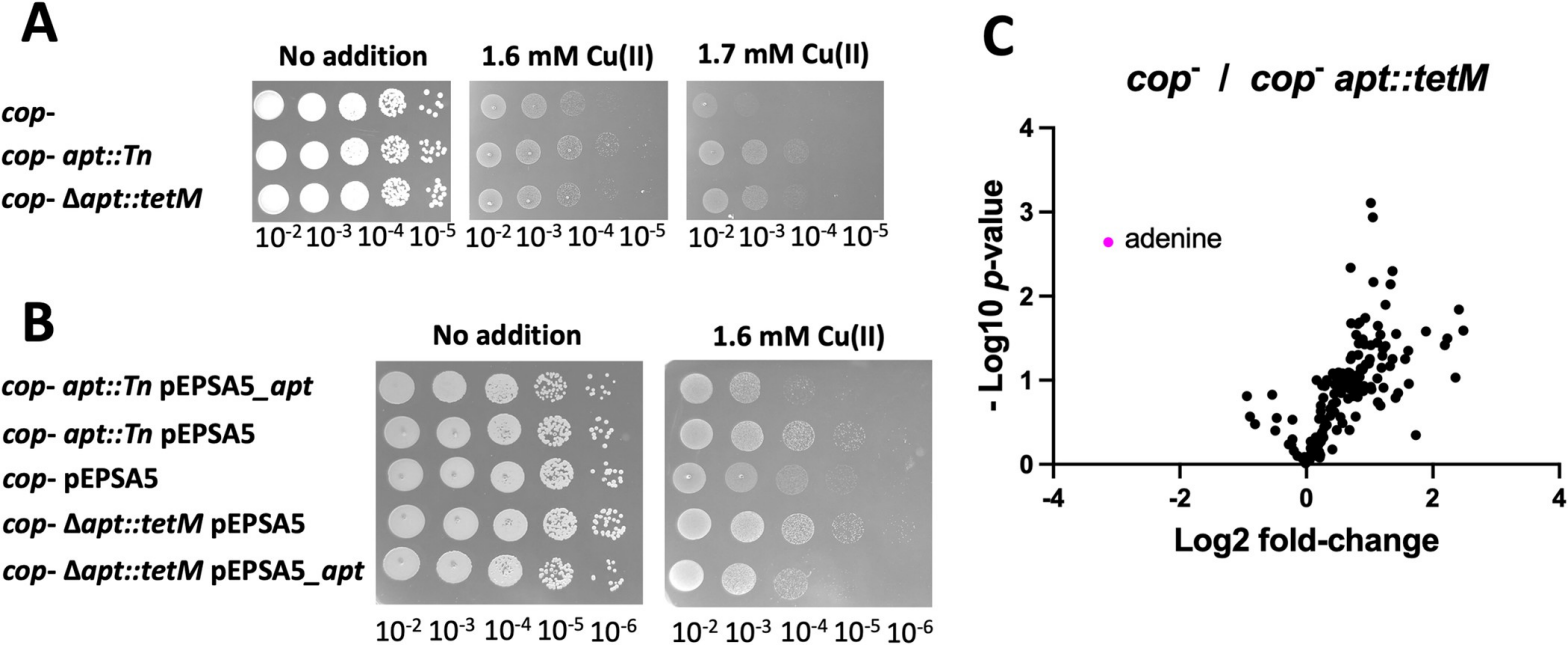
A null mutation in *apt* suppresses the Cu(II) sensitivity of the *cop*^-^ strain. Panel A, overnight cultures of the *cop*^*-*^, *cop*^*-*^ *apt*::*Tn*, and *cop*^*-*^ Δ*apt*::*tetM* strains were serially diluted and spot plated on TSA media with 0, 1.6, and 1.7 mM Cu(II). Panel B, overnight cultures of the *cop*^-^, *cop*^*-*^ *apt*::*Tn*, and *cop*^*-*^ Δ*apt*::*tetM* strains containing the pEPSA5_*apt* or pEPSA5 (empty vector) were serially diluted and spot plated onto TSA chloramphenicol media with 0 or 1.6 mM Cu(II). Panel C, Volcano plots of Log2 fold change of metabolite abundances isolated from *cop*^-^ divided by those of the *cop*^-^ *apt*::*tetM* strain. Data represent the average of three biological replicates. Photos of representative experiments displayed.

To verify the function of Apt (SAUSA300_1591) in protecting against Cu(II) intoxication, we created an Δ*apt*::*tetM cop*^*-*^ strain. The Δ*apt*::*tetM* mutation provided resistance to Cu(II) when compared to the *cop*^*-*^ strain in solid or liquid media containing Cu(II) (Figs 4A and S3). Since *apt* appears to share a promoter with *recJ*, we cloned *apt* into a vector (pEPSA5) that placed it under a non-native promoter (*xylRO*). Introduction of the pEPSA5_*apt* vector into the *cop*^*-*^ *apt*::*Tn* or *cop*^*-*^ Δ*apt*::*tetM* strain returned growth to that of the *cop*^*-*^ strain containing empty vector when Cu(II) was present (Fig 4B). Note that it was not necessary to induce expression of *apt* and the basal “leaky” expression was sufficient to complement the mutants.

We next tested the hypothesis that the absence of Apt has no effect in intracellular Cu accumulation. To this end, the *cop*^*-*^ Δ*apt*::*tetM* and *cop*^*-*^ strains were cultured in TSB for 8 hours before adding 5 μM Cu(II) and incubated for an additional 60 minutes. We determined the concentration of cell associated Cu using ICPMS analysis and standardized the amount of Cu associated with the cells to the concentration of magnesium or sulfur. Both strains accumulated the same amount of Cu (S4 Fig).

The iron-sulfur cluster containing dehydratase aconitase (AcnA) is inactivated by Cu(I) [6]. We previously found that strains that have decreased Cu ion uptake protected AcnA from Cu(I) poisoning. The absence of Apt did not alter the sensitivity of AcnA to Cu ions which is consistent with the ICPMS data in suggesting that the lack of Apt does not alter Cu ion uptake or intracellular Cu ion accumulation (S5 Fig). Taken together, these data led to the hypothesis that the absence of Apt reroutes metabolism to promote Cu(II) resistance.

The *apt* gene is predicted to code for an adenine phosphoribosyltransferase that functions in purine salvage [35]. Apt catalyzes the conversion of adenine (or guanosine) and phosphoribosyl pyrophosphonate (PRPP) to AMP (or GMP) and pyrophosphate [35]. We used untargeted metabolomic analysis to examine whether Apt functioned in adenine homeostasis in *S*. *aureus*. The *cop*^-^ Δ*apt*::*tetM* strain accumulated 10-fold more adenine than the *cop*^-^ strain verifying a role for Apt in adenine homeostasis (Fig 4C and Tables 3 and S3). The *cop*^*-*^ Δ*apt*::*tetM* strain had decreased accumulation of metabolites of the pentose phosphate pathway (R5P, sedoheptulose), purine precursors (AICAR, AICA-riboside), and pyrimidines (dUMP, cytosine) verifying that it’s absence is results in a metabolic reprograming of nucleotide metabolism.

**Table 3:** Significantly altered metabolites in the *cop*^*-*^ Δ*apt*::*tetM* strain.^a^ *.

*cop*^-^ / *cop*^*-*^ Δ*apt*	
Metabolite	Log2 fold-change
Adenine	-3.1
AICAR (ZMP)	2.2
AICA-riboside	0.8
Cytosine	1.0
dUMP	1.9
Lactate	1.2
Mevalonate	2.2
Orotate^b^	>10
Ribose 5-phosphate	1.6
Sedoheptulose	2.4
UDP-Glucose	0.9

^a^ A paired student’s t-test was run and metabolites with a p <0.05 displayed.

^b^ Orotate was below the detectable limit upon Cu addition.

* A full list of metabolites can be found in S3 Table.

### Genetic evidence suggests that modulating PRPP levels alters growth in the presence of Cu(II)

**Fig 5:**
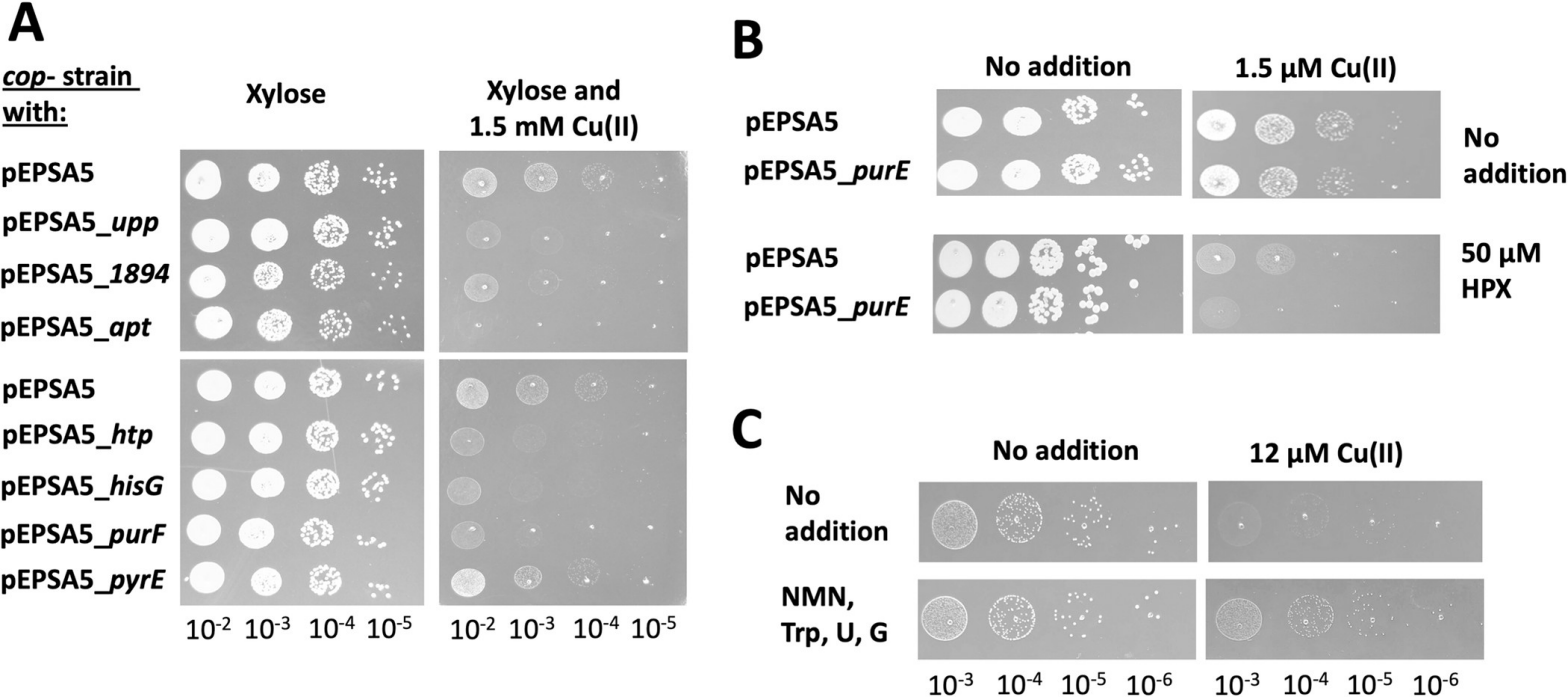
Altering the demand for PRPP modulates the sensitivity to Cu(II). Panel A, cultures of the *cop*^-^ strain containing the empty vector (pEPSA5) or plasmid that over-expresses a gene coding a PRPP utilizing enzyme were serial diluted and spot plated on solid TSA chloramphenicol media containing 0.5% xylose and 0 or 1.5 mM Cu(II). Panel B, cultures of the *cop*^-^ strain containing the empty vector (pEPSA5) or plasmid that over-expresses *purE* were serial diluted and spot plated on solid TSA chloramphenicol media containing 0.5% xylose, 0 or 1.5 mM Cu(II), and with or without 50 μM hypoxanthine (HPX). Panel C, cultures of the *cop*^-^ strain were serial diluted and spot plated on defined glucose medium with 0 or 12 μM Cu(II) and without and with 50 μM uridine (U), tryptophan (Trp), guanosine (G), and nicotinamide mononucleotide (NMN). Photos of representative experiments displayed.

We hypothesized that increasing Apt activity would decrease PRPP levels resulting in increased sensitivity to Cu(II). To this end, we overexpressed *apt* using pEPSA5_*apt* and added xylose to induce expression. The *cop*^*-*^ strain containing pEPSA5_*apt* was significantly more sensitive to Cu(II) than the *cop*^*-*^ strain containing the empty vector (pEPSA5) (Figs 5A and S6).

In *S*. *aureus*, there are nine enzymes that utilize PRPP as a co-substrate (Table 4) [36–38]. We hypothesized that overproduction of any enzyme that utilizes PRPP will increase in Cu(II) sensitivity. We created eight plasmid vectors (including the *apt* clone) using pEPSA5 that allowed us to induce transcription of the individual genes. When cultured on TSA with xylose, none of the strains over-producing PRPP utilizing enzymes displayed a growth phenotype. However, if TSB medium was supplemented with Cu(II) and xylose, strains over-producing *apt*, *upp*, *htp*, *hisG*, *purF*, or SAUSA300_1894 displayed worse growth than the *cop*^*-*^ strain carrying the empty vector (Fig 5A). Overproduction of *purE* or *pyrE* did not alter growth in the presence or absence of Cu(II) in TSB medium. However, when cultured on defined medium supplemented with hypoxanthine (HPX) or orotate, the co-substrates for PurE, the *cop*^-^ strain over-expressing *purE* was sensitized to Cu(II) (Figs 5B and S7A). Likewise, the inclusion of HPX in the presence of Cu(II) increased the growth defect of the strain over-expressing *hpt* (S7B Fig). Adding the substrates for SAUSA300_1804 (nicotinate), Upp (uridine), or Apt (adenine) sensitized the *cop*^*-*^ strains to growth in the presence of Cu(II) (S7 Fig). For these experiments we did not add xylose to the growth media and relied on the “leaky” *xylRO* promoter, which is why there is less of an effect with Cu(II) where compared to the data displayed in Fig 5A.

**Table 4:** *S*. *aureus* PRPP utilizing enzymes.

Enzyme	Reaction	Name	Locus tag
Ribose-phosphate pyrophosphokinase	AMP + PRPP = ribose 5-phosphate + ATP	Prs	SAUSA300_0478
Nicotinate phosphoribosyltransferase	nicotinate + PRPP + ATP = β-nicotinate D-ribonucleotide + PP_*i*_ + ADP + P_*i*_		SAUSA300_1894
Adenine phosphoribosyltransferase	PRPP + adenine = AMP + PP_*i*_	Apt	SAUSA300_1591
ATP phosphoribosyltransferase	PRPP +ATP = 1-(5-phospho-β-D-ribosyl)-ATP + diphosphate	HisG	SAUSA300_2612
Hypoxanthine-guanine phosphoribosyltransferase	hypoxanthine + PRPP = inosine monophosphate guanine + PRPP = guanosine monophosphate.	Hpt	SAUSA300_0488
Uracil phosphoribosyltransferase	Uracil + PRPP = UMP	Upp	SAUSA300_2066
Anthranilate phosphoribosyltransferase	Anthranilate + PRPP = N-(5-phospho-D-ribosyl)-anthranilate + PP_*i*_	TrpD	SAUSA300_1264
Orotate phosphoribosyltransferase	Orotate + PRPP = orotidine 5’-phosphate + PP_*i*_	PyrE	SAUSA300_1098
Amidophosphoribosyltransferase	PRPP + H_2_O + glutamine = 5-phospho-β-D-ribosylamine + diphosphate + glutamate	PurF	SAUSA300_0972
Xanthine phosphoribosyltransferase	XMP + diphosphate = 5-phospho-alpha-D-ribose 1-diphosphate + xanthine	Xpt	SAUSA300_0386

We tested the hypothesis that providing metabolites that require PRPP for synthesis will improve growth in the presence of Cu(II). We supplemented defined growth medium with uridine, tryptophan, guanosine, and nicotinamide mononucleotide (NMN) which have been shown to partially bypass the need for PRPP synthesis [39]. The addition of these compounds did not have a significant effect on the growth of the *cop*^-^ strain in the absence of Cu(II); however, the presence of these compounds improved growth of the *cop*^-^ strain if the medium was supplemented with Cu(II) (Figs 5C and S8).

### Altering *prs* expression modulates sensitivity to Cu(II)

Prs phosphorylates R5P using ATP to produce PRPP and AMP [36]. The findings that R5P accumulated upon growth with Cu(II) and that altering PRPP levels changed sensitivity to Cu(II) suggested that Prs is inhibited by Cu ions. Increasing or decreasing the abundance of an enzyme can increase or decrease resistance to an inhibitor, respectively [40, 41]. We tested the hypothesis that modulating *prs* expression will alter the Cu(II) sensitivity phenotype of the *cop*^-^ strain.

We developed a plasmid based CRIPSRi system that utilizes the “dead” Cas9 (dCas9) from *Streptococcus pyogenes* to decrease the transcription of genes [42, 43]. We used pSK as the vector backbone, which is a low copy plasmid in *S*. *aureus* [44]. The guide RNAs were constitutively expressed and *dcas9* was under the transcriptional control of *tetRO*. We chose two different regions on *prs* that contain PAM sequences to target with guide RNAs (*prs1* and *prs2*). We included two control strains for the experiment. The first control strain contained a vector with a guide RNA that was not homologous to the USA300_LAC genome [43] and the other with a guide RNA targeted to *hla*, which codes for alpha hemolysin [43].

The *cop*^*-*^ strain containing the CRISPRi system with guide RNAs specific to *prs*, *hla*, or the non-homologous guild RNA were plated on TSB media with or without 100 ng mL^-1^ anhydrotetracycline (Atet). Plating with Atet resulted in death of the *cop*^*-*^ strains containing the *prs* guide RNAs but not the strains containing the *hla* or non-homologous guide RNAs (S9 Fig). These data are consistent with previous studies suggesting that *prs* being an essential gene in *S*. *aureus* [45].

**Fig 6:**
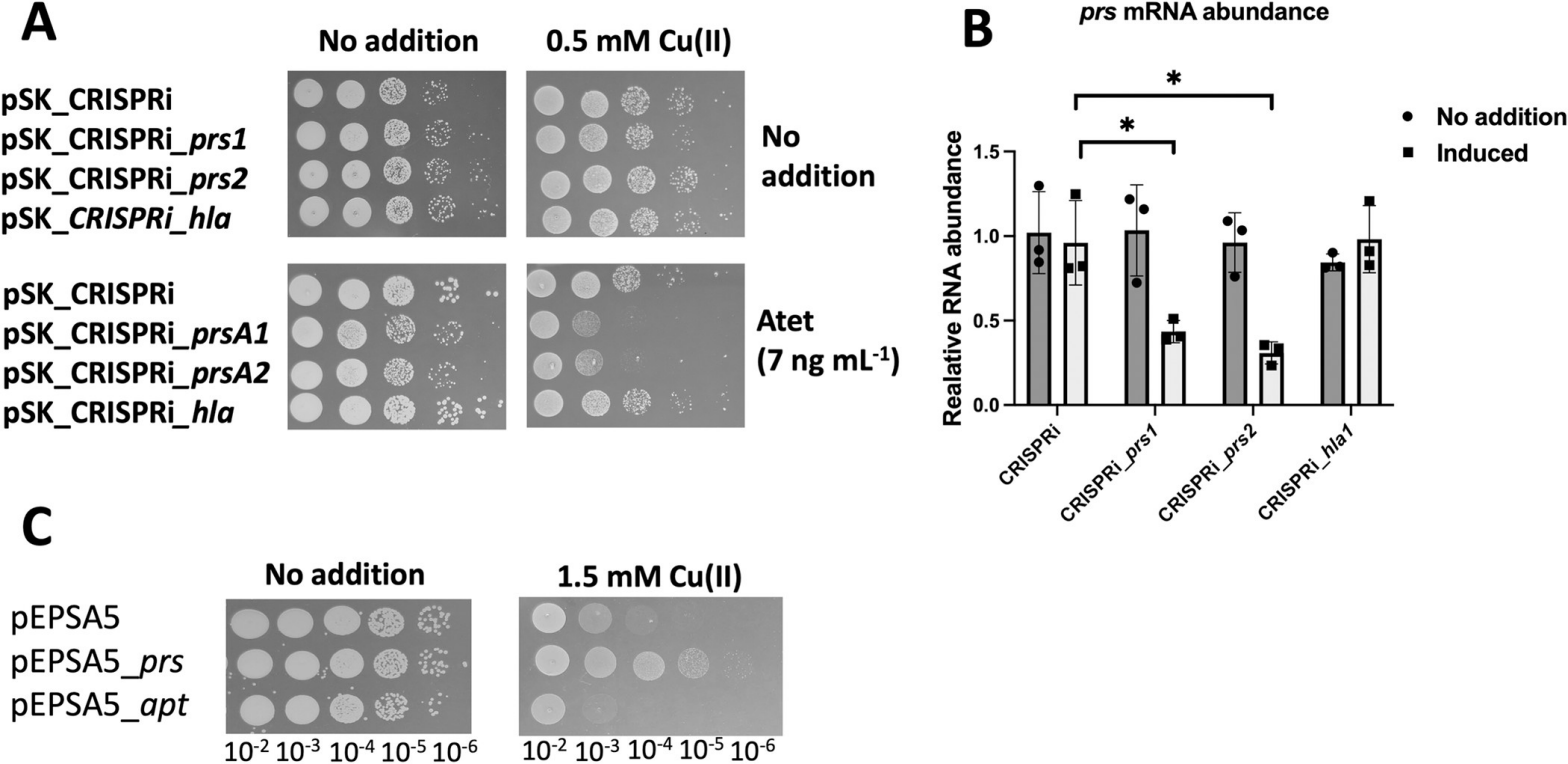
Modulating *prs* expression alters sensitivity to Cu(II). Panel A, cultures of the *cop*^-^ strain containing a pSK_CRIPSRi vector were serial diluted and spot plated on TSA chloramphenicol media with 0 or 0.5 mM Cu(II) with or without 7 ng mL^-1^ anhydrotetracycline (Atet) to induce *dcas9* expression. The gene targeted by the sgRNA are displayed except for the control which contains a randomized sgRNA (pSK_CRIPSRi). Panel B, the abundances of RNAs corresponding to *prs* were quantified from the same strains listed in Panel A after culture in TSB chloramphenicol media with (dark bars) and without Atet (light bars). Panel C, cultures of the *cop*^-^ strain containing a pEPSA5 (empty vector), pEPSA5_*prs*, or pEPSA5_*apt* were serial diluted and spot plated on TSA chloramphenicol medium with 0 or 1.5 mM Cu(II). The data in Panel B represent the average of three biological replicates and standard deviations are displayed. Student’s t-tests were performed on the data and * indicates p < 0.05. Photos of representative experiments displayed.

We next plated the strains on TSA containing a lower concentration of Atet as to decrease the stringency of the CRISPRi-based inhibition and examined sensitivity to growth with Cu(II). When Atet was included in the medium, the *cop*^*-*^ strains containing the *prs* guide RNAs displayed a hyper-sensitivity to growth in the presence of Cu(II) when compared to the *cop*^*-*^ strains containing the *hla* guide RNA or the empty vector (Fig 6A). The concentration of Atet utilized did not result in a growth defect on TSB medium and the concentration of Cu(II) included did not result in cell death if Atet was not included.

Two experiments were used to ensure that our CRIPRi system was working properly. First, we spot plated the *cop*^*-*^ strain containing the *hla*, *prs*, or non-homologous guide RNAs on TSA media containing 5% rabbit blood. The addition of Atet resulted in decreased hemolysis in the strain containing the *hla* guide RNA but not the strains expressing the *prs1* or non-homologous guild RNAs (S10A Fig). We next quantified RNA transcripts corresponding to *hla* and *prs*. The *hla* and *prs* transcripts were significantly decreased upon induction with Atet in strains containing the corresponding guide RNAs (Figs 6B and S10B).

We increased the gene dosage of *prs* by placing it under the transcriptional control of *xylRO* using pEPSA5. For these experiments we did not add xylose to the growth media and relied on the “leaky” *xylRO* promoter for expression. The *cop*^*-*^ strain containing pEPSA5_*prs* grew more proficiently than the *cop*^*-*^ strain containing pEPSA5 on solid or liquid media medium containing Cu(II) whereas there was not an effect on TSB medium (Figs 6C and S6). We included the *cop*^-^ strain containing pEPSA_*apt* as a control. Taken together, these data are consistent with the hypothesis that increasing or decreasing *prs* transcription results in hypo- and hyper-sensitivity to Cu(II) in the growth media, respectively.

### Copper inhibits Prs

**Fig 7:**
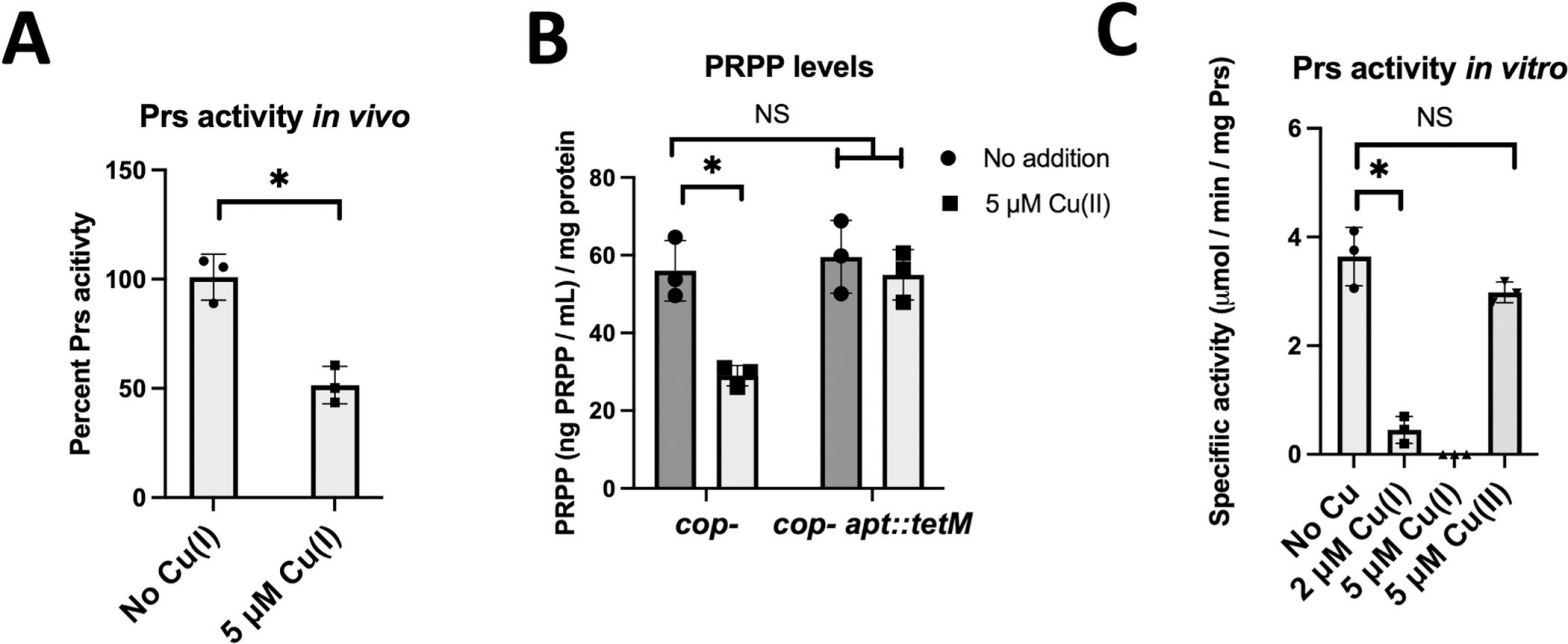
Cu ions inhibit Prs. Panel A, Prs activity was determined in cell-free lysates from the *cop*^-^ strain after culture in TSB chloramphenicol medium with 1% xylose and with 0 or 5 μM Cu(II). Panel B, PRPP levels were determined in cell lysates from the *cop*^-^ strain after growth in TSB medium without (dark bars) and with 5 μM Cu(II) (light bars). Panel C, activity of Prs was monitored in vitro with and without Cu ions. The data displayed are the average of three biological replicates (Panels A and B) or biochemical experiments (Panel C) and standard deviations are displayed. Student’s t-tests were performed on the data and * indicates p < 0.05.

We tested the hypothesis that Cu ions inhibit Prs. To measure Prs activity we used a coupled assay in which the AMP produced from the ATP-dependent phosphorylation of R5P is measured using an NADH-coupled enzyme system [46]. This assay was unable to detect an increase in R5P-dependent AMP production (above background levels) in cell-free lysates generated from the *cop*^-^ strain. To circumvent this problem, we utilized the *cop*^-^ strain containing pEPSA5_*prs* which allowed us to over-produce Prs. Uncoupling *prs* transcription from its native promoter also decreased the likelihood that growth in the presence of Cu(II) alters *prs* promoter activity. R5P-dependent AMP production could be detected in cell-free lysates generated from the *cop*^-^ pEPSA5_*prs* strain (Fig 7A). We next monitored Prs activity in the *cop*^*-*^ pEPSA5_*prs* strain after growth in TSB-xylose media the presence or absence of Cu(II). The R5P-dependent AMP generation was decreased by 40% in cell lysates from cells that were exposed to 5 μM Cu(II) (Fig 7A).

We tested the hypothesis that PRPP levels would be decreased upon growth in the presence of Cu(II). We cultured the *cop*- and *cop-* Δ*apt*::*tetM* strains in the presence and absence 5 μM Cu(II) and quantified PRPP levels in cell-free lysates. PRPP levels were approximately 50% in the *cop-* strain co-cultured with Cu(II) (Fig 7B). Surprisingly, the PRPP levels in the *cop*^*-*^ Δ*apt*::*tetM* strain were the same as found in the *cop*^-^ strain and addition of Cu(II) had no effect on PRPP levels, suggesting a mechanism for how the null *apt* mutations suppress the Cu resistance of the *cop*^*-*^ strain.

We next purified recombinantly produced Prs and assessed activity in the presence and absence of Cu ions. Once Cu(II) enters the cytosol it interacts with thiol containing compounds which aid its reduction to Cu(I) [6, 47]. The addition of 2 μM Cu(I) resulted in a 7-fold decrease in Prs activity and activity could not be detected after the addition of 5 μM Cu(I) (Fig 7C). The addition of 5 μM Cu(II) did not result in a significant decrease in Prs activity. We left Prs out of the assay mix and examined the effect of 5 μM Cu(I) on the coupling enzyme mixture. Cu(I) did not inhibit AMP-dependent NADH oxidation, suggesting that, at the concentrations tested, the Cu(I) ions were not significantly inhibiting the coupling enzymes, but rather inhibiting Prs (S11 Fig).

### Removing host derived Cu ions from the cytosol is important for pathogenesis

**Fig 8:**
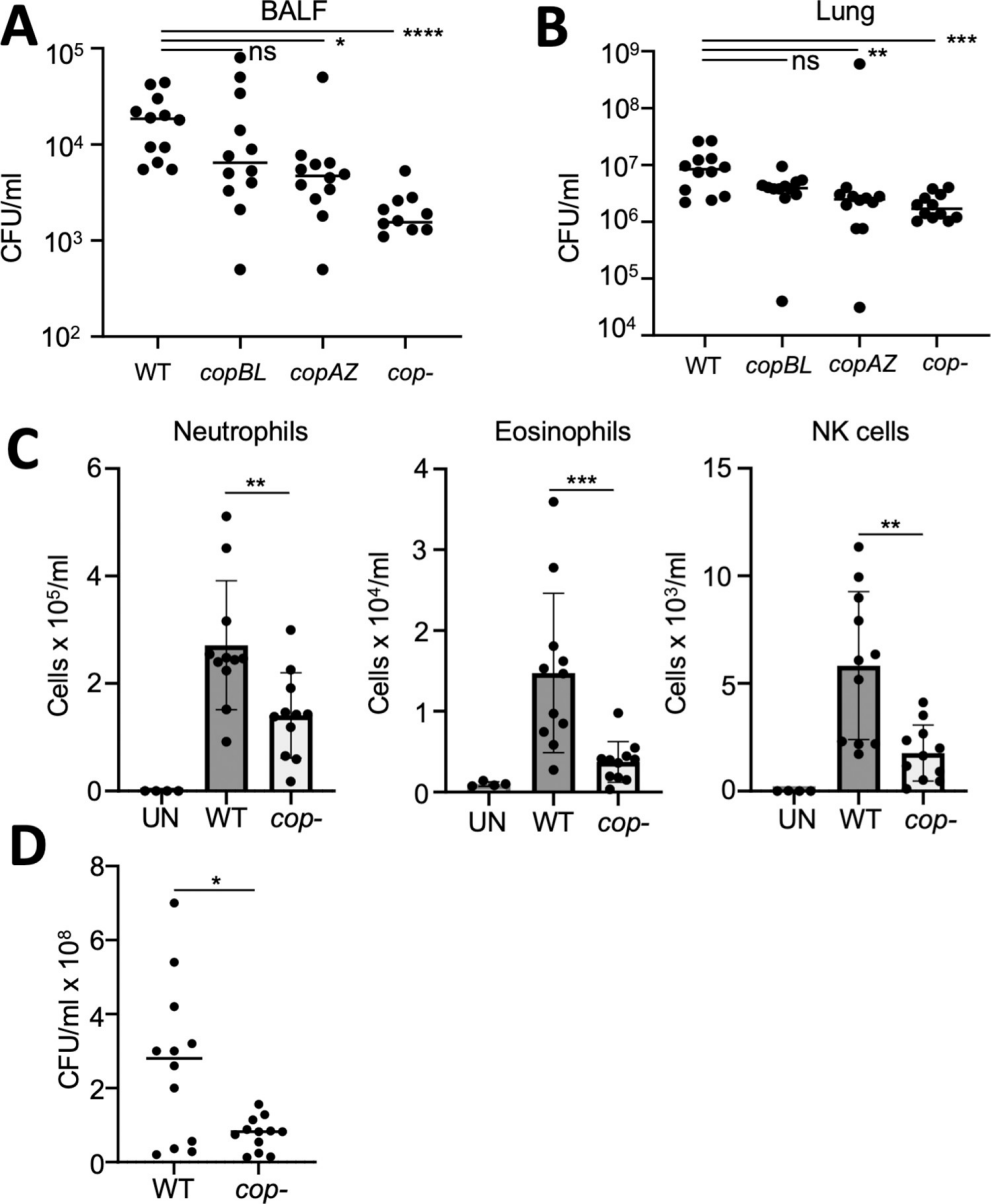
Copper detoxification contributes to *S*. *aureus* pathogenesis. C57BL/6 mice were intranasally infected with *S*. *aureus* USA300 and copper transport mutants for 24 h before euthanasia. A) Bacterial counts in BALF. B) Bacterial counts in lung tissue. C) Flow cytometric quantification of recruited immune cells to the airway in BALF. D) C57BL/6 mice were subcutaneously infected with *S*. *aureus* USA300 and copper transport mutant for five days before euthanasia. Each point represents a mouse. Lines display median. Bar graphs displays means with standard deviation. UN-uninfected, ns-not significant, *p<0.05, **p<0.01, ***p<0.001 and ****p<0.0001.

Work by others has demonstrated that effective removal of Cu ions from the cytosol is important for survival when challenged with human blood or macrophages [22]. Macrophages play an important role in clearing *S*. *aureus* from lung and bronchoalveolar lavage fluid, and thereby, protect against infection [48, 49]. We examined the pathogenesis phenotypes of the *cop*^*-*^ strain and the individual copper-export mutants in a murine model of acute pneumonia. A small but insignificant decrease in bacteria in the BALF was observed with the Δ*copBL* mutant. A 58% reduction in bacterial burden in BALF was detected with the Δ*copAZ* strain, while the *cop*^*-*^ strain saw a 91% reduction (*p*<0.0001; Fig 8A). This pattern of clearance was also observed in lung tissue (Fig 8B). Significantly fewer neutrophils, eosinophils, and NK cells were detected in the BALF, likely due to the reduced bacterial burden (Fig 8C). The attenuation of the *cop*^-^ strain was further confirmed in a murine model of skin and soft tissue infection. By day 5 of infection, the *cop*- strain had 78% less (Fig 8D) bacteria than the WT strain. These data confirm the important role Cu detoxification plays in the pathogenesis of *S*. *aureus* infection.

## Discussion

The inhibition of enzymes by Cu ions has been demonstrated, but mechanism(s) by which Cu ions kill or inhibit the growth of bacteria remains unknown and is likely multifaceted. Because of this, it has been difficult for researchers to identify a single cellular target that when inhibited by Cu results in cell death or growth inhibtion. It was demonstrated that Cu(I) can cause the displacement of solvent accessible FeS clusters in proteins, such as certain SAM radical proteins or dehydratases, yielding an inactive catalyst in enzymes that require the cofactor for function [6, 50]. However, the roles of the described dehydratases and SAM-radical enzymes can be bypassed by growth in a rich medium such as Luria-Bertani broth.

Additional work in *E*. *coli* suggests that the A-type FeS cluster carriers SufA and IscA, as well as the FeS cluster synthesis scaffold IscU, bound Cu(I) *in vivo* and are likely inhibited in FeS protein assembly and/or synthesis when associated with Cu [9, 11]. *E*. *coli* utilizes two FeS cluster synthesis systems, ISC and SUF, that share a degree of functional, but not biochemical, redundancy [51]. The deletion of both *sufA* and *iscA* is synthetically lethal [52]. Therefore, complete inhibition of the functions of SufA and IscA (or ErpA) by Cu(I) should result in death. *S*. *aureus* utilizes the SUF system to synthesize FeS clusters [53]. Deletion of *sufA* has little to no measurable impact on *S*. *aureus* fitness suggesting that Cu ion inhibition of A-type scaffold is not lethal [54].

Additional described effects of Cu ions on physiology may include ROS generation, inhibition of cytochrome oxidase, and increasing protein aggregation. Imlay and colleagues have presented evidence that cytosolic Cu ions likely do not directly contribute to ROS generation in *E*. *coli* [15]. Cytochrome oxidases are not essential in *S*. *aureus* [55]. It was recently demonstrated that when *E*. *coli* is cultured anaerobically in the presence of Cu(I) ions there is an increase in protein aggregation under anaerobic growth [56]. The authors did not report significantly increased protein aggregation during aerobic growth conditions and at low copper concentrations, such as those described in this study.

We demonstrate that there is a large variation in the amount of Cu(II) that is required to inhibit growth of the *cop*^-^ strain if the media is varied. Low μM concentrations of Cu(II) are required for growth inhibition in chemically defined media whereas mM concentrations are required in complex media. Defined media contains fewer molecules, such as amino acids, that can interreact with metal ions including Cu in making the Cu ions more bioavailable [57]. In addition, Cu ions interrupt numerous metabolic processes (glycolysis, pentose phosphate pathway, TCA cycle, FeS cluster synthesis, etc.) and metabolites present in complex media may allow for bypass of some of these metabolic bottlenecks.

**Fig 9:**
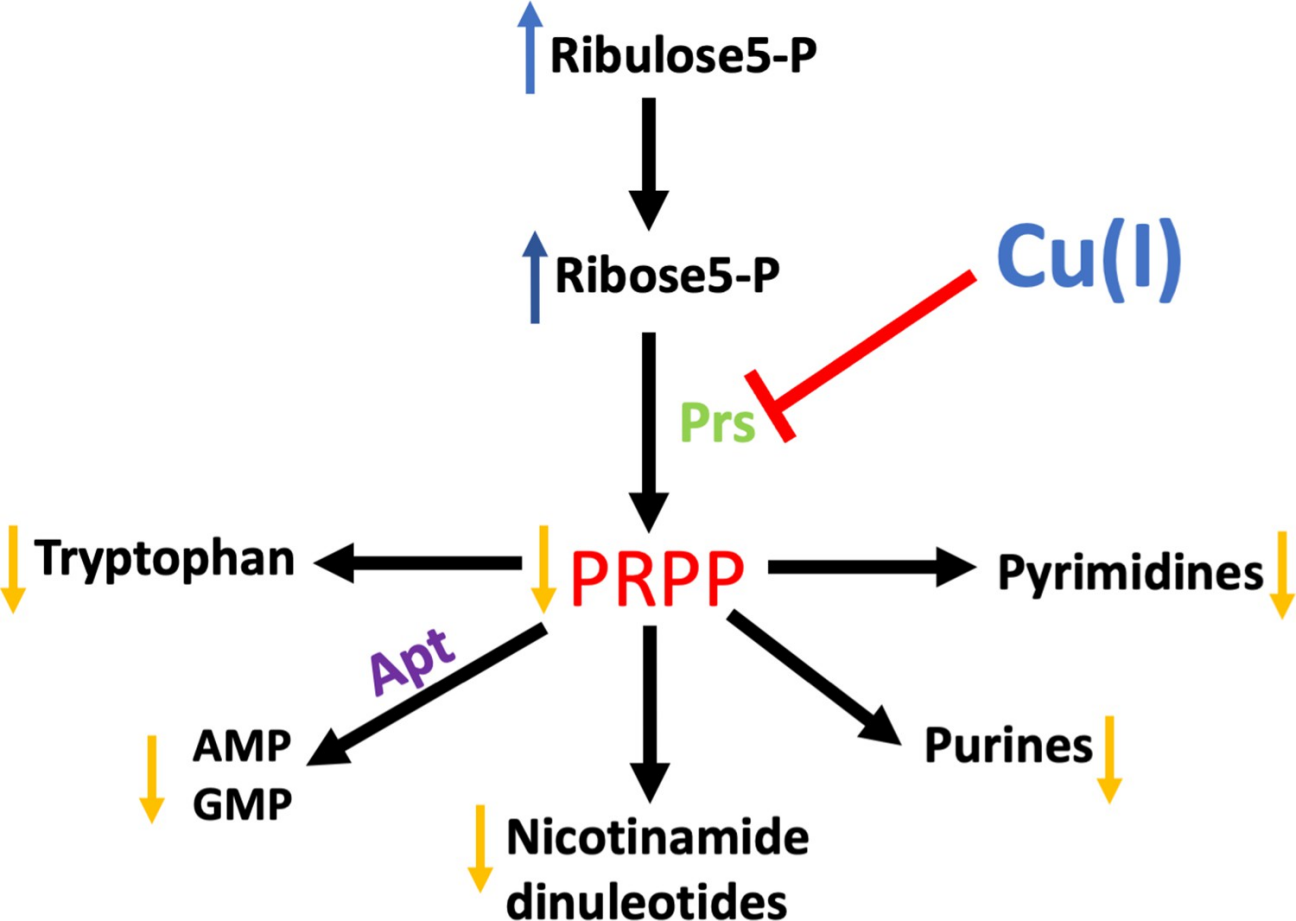
Model for metabolic effects of inhibition of Prs by Cu(I) in *Staphylococcus aureus*.

The data presented herein support a model where growth in the presence of Cu ions results in the inhibition of the essential enzyme Prs (Fig 9). In support of this model, glycolytic and PPP metabolites accumulate upon Cu(II) treatment, whereas PRPP levels decrease. Physiological or genetic alterations that vary carbon flux though the PPP altered sensitivity to Cu(II). Modulating PRPP pools or bypassing the need for PRPP synthesis by adding metabolites that require PRPP for synthesis altered sensitivity to Cu(II). Increasing or decreasing *prs* expression improved and impaired growth with Cu(II), respectively. Lately, Prs activity was decreased in cell lysates after growth with Cu(II) and Prs was inhibited *in vitro* by Cu(I).

It is currently unclear how Cu(I) is inhibiting Prs. Prs does require a divalent metal and it prefers Mg(II) or Mn(II) [36]. It was reported that Ca(II) acts as an inhibitor of the *Salmonella enterica* Prs by preventing Mg(II) binding [36]. Similar as reported here, Prs was not inhibited by Cu(II) *in vitro*, but the authors did not examine Cu(I) [58]. We note that there is a conserved histidine residue in the active site of Prs that is utilized for coordinating the ribose [59, 60]. We theorize that Cu(I) inactivates Prs by blocking active site access or by mismetalating the enzyme, and we plan to test these hypotheses in the future.

This manuscript complements a previous study demonstrating that Cu ions inhibit glyceraldehyde 3-dehydrogenase (GapDH) [61]. A proteomic study found that several enzymes involved in glycolysis and the PPP (ex. Fda, GapA, Tkt) had increased abundances upon growth with Cu(II). It is likely that these alterations were the result of Cu ions altering the ability to flux carbon through these pathways. The only two operons in *S*. *aureus* strain LAC that that been demonstrated to respond to the Cu-dependent transcription factor CsoR are *copAZ* and *copBL* suggesting that the altered expression of the genes that encode these metabolic enzymes are not responding directly to the presence of Cu [62, 63]. Studies have found that GapA binds to Cu ions *in vivo* and *in vitro* [61, 64]. The metabolomic data presented here found that several glycolytic intermediates were increased when cells were cultured with Cu(II) for 30 minutes. We also noticed accumulation of 3-phosphoglycerate which is the product of GapA suggesting that there maybe also metabolic bottleneck downstream of GapA. Glyceraldehyde 3-phosphate, fructose 6-phosphate, and glucose 6-phosphate are shared metabolites between glycolysis and PPP, so, it would be expected that there would be altered abundances of these metabolites if PPP function was decreased.

Cu ions are used by human macrophages to enhance bacterial clarence and several studies have highlighted the importance for proper Cu ion detoxification for *S*. *aureus* pathogenesis [21]. For the most part, studies have examined the role of the second horizontally transferred Cu(I) detoxification system CopBL transporter which is ubiquitous in USA300 linage. The *copBL* operon is encoded in the ACME transposable element [27]. Zapotoczna et al. examined the role of the *copBL* for survival when challenged with macrophages [22]. *S*. *aureus* lacking CopB or CopL had significantly decreased survival in murine macrophages whereas a *copA* mutant had similar survival as the parent strain. It was also demonstrated that a strain carrying an allele of *csoR* (CHC strain) that encode a protein with decreased capability to respond to Cu(I) has decreased ability to survive in mouse macrophages affirming the importance of detecting and responding to Cu. The authors also noted an increased ability of a strain with two copper detoxification systems to survive in human whole blood. Likewise, the CHC strain showed decreased whole blood survival compared to the wild-type strain [22]. In a separate study, Purves et al. demonstrated that Δ*copB* or Δ*copL* strains had decreased survival in murine macrophages [23]. The concentration of Cu was elevated in urine from patients experiencing an infection of the urinary tract [24]. Using a murine urinary tract model of infection, the WT strain was competed against a Δ*copB* or Δ*copL* mutant and determined that both genes contribute to fitness. The Δ*copB* mutant had decreased colonization of urinary tracts compared to the parental strain [24]. The studies presented herein did not discern a phenotype for the Δ*copBL* mutant in the lungs or the BALF; however, our findings did highlight a role for *copAZ* and the general export of Cu ions in lung pathogenesis. Moreover, we determined that Cu detoxification has a role in survival in a skin model of infection.

The data presented in this manuscript improves our knowledge of how intracellular Cu ions toxify cells. It also provides a mechanism by which Cu ions inhibit the growth of *S*. *aureus*. We demonstrated that Cu ion detoxification is necessary for survival in murine BALF and lung tissue in a pneumonia model of infection, as well as in a murine model of skin and soft tissue infection. We have devised a working model wherein Cu(I) inhibits PPP function resulting in decreased levels of the essential metabolite PRPP (Fig 9). It is yet to be determined whether the inhibition of the PPP by Cu ions is resulting in a decreased capability of *S*. *aureus* to cause infection.

## Materials and methods

### Ethics statement

Animal work in this study was carried out in strict accordance with the recommendations in the Guide for the Care and Use of Laboratory Animals of the NIH (National Academies Press, 2011), the Animal Welfare Act, and US federal law. Protocols were approved by the Institutional Animal Care and Use Committee of Rutgers New Jersey Medical School of Newark, New Jersey, USA.

## Dryad DOI

https://doi.org/10.5061/dryad.8w9ghx3s0 [76]

### Chemicals and bacterial growth conditions

All bacterial strains used for the studies were derived from the community associated *S*. *aureus* MRSA strain USA300_LAC [65]. Strains were grown at 37°C in tryptic soy broth (TSB) (MP Biomedicals) or a chemically defined medium containing 11 mM glucose or 11 mM gluconate as a primary carbon source [66]. Solid tryptic soy (TSA) or chemically defined media was generated by adding 1.5% (weight/vol) agar (VWR). Liquid cultures were shaken at 200 rpm. Chelex-treated TSB was prepared as previously described [31]. Unless stated otherwise, cells were cultured in 10 mL capacity culture tubes containing 1.5 mL of liquid medium. The restriction minus strain *S*. *aureus* RN4220 was used for transformation [67] and transductions were done using bacteriophage 80α [68]. All *S*. *aureus* strains (Table 5) used in this study were derived from the Δ*copAZ* Δ*copBL* mutant (*cop*^*-*^ strain) [31]. *Escherichia coli* DH5α was used for plasmids preparation (NEB) and *E*. *coli* BL21 was used for protein purification. Both were cultured in lysogeny broth.

**Table 5:** Strains and plasmids used in this study.

Microbial strains utilized in this study		
Name	Chromosomal Genotype^a^	Reference
***S*. *aureus* USA300 LAC strains**		
JMB 8573	Δ*copBL* Δ*copAZ* (*cop*^-^)	[31]
JMB 7901	Δ*copBL*	[5]
JMB 8571	Δ*copAZ*	[31]
JMB 8902	*cop*^*-*^ *apt*::*Tn* (*ermB*) (SAUSA300_1591)	[31]
JMB 9138	*cop*^*-*^ *Δapt*::*tetR* (SAUSA300_1591)	This study
JMB 10462	*cop*^*-*^ *pglS*::*Tn*(*ermB*)(SAUSA300_1902)	This study and [77]
JMB 11379	*cop*^*-*^ *Δtkt*::*kan* (SAUSA300_1239)	This study and [78]
JMB 9535	*cop*^*-*^ pLL39	[32]
JMB 13670	*cop*^*-*^ *pglS*::*Tn* pLL39	This study and
JMB 13672	*cop*^*-*^ *pglS*::*Tn* pLL39_pglS	This study and
**Other strains**		
RN4220	Restriction minus *S*. *aureus*	[67]
DH5α	Restriction minus *E*. *coli*	
BL21 AI*	Expression *E*. *coli*	
FY2	*S*. *cerevisiae* for YCC	[79]
**Plasmids used in this study**		
**Name**	**Function**	**Reference**
pJB38	Construction of gene deletions	[80]
pEPSA5	XylR dependent transcription	[81]
pSK	Shuttle vector	[82]
pSK_CRISPR	Expression of CRISPR/dCas9 system	[43] and this study
pSK_CRISPR_*hla*	CRISPR/dCas9 directed at *hla*	[43] and this study
pSK_CRISPR_*prsA1*	CRISPR/dCas9 directed at *prsA*	This study
pSK_CRISPR_*prsA2*	CRISPR/dCas9 directed at *prsA*	This study
pEPSA5_*apt*	*ap*t under xylose inducible promoter	This study
pLL39	Genetic complementation	[83]
pLL39_*pglS*	*pglS* under native promoter	This study
pEPSA5_*prsA*	*prsA* under xylose inducible promoter	This study
pEPSA5_*hpt*	*hpt* under xylose inducible promoter	This study
pEPSA5_*hisG*	*hisG* under xylose inducible promoter	This study
pEPSA5_*purF*	*purF* under xylose inducible promoter	This study
pEPSA5_*pyrE*	*pyrF* under xylose inducible promoter	This study
pEPSA5_*1894*	*1894* under xylose inducible promoter	This study
pGEX-6P-1_*prsA*	Expression of Prs for purification	This study
pJB38_Δ*apt*::*tetM*	Generate *Δapt*::*tetM* allele	This study

^a^Abbreviations: *Tn*, transposon

Antibiotics were added at the final following concentrations: 100 μg mL^-1^ ampicillin (Amp); 50 μg mL^-1^ Kanamycin (Kan); 10 μg mL^-1^ chloramphenicol (Cm); 10 μg mL^-1^ to select for plasmids and 3.3 μg mL^-1^ to maintain plasmids; erythromycin (Erm); 3 μg mL^-1^ tetracycline (Tet); 3–100 ng mL^-1^ anhydrotetracycline (Atet). The defibrinated rabbit blood agar plates were made as previously described [69]. The CuSO_4_ was prepared in deionized and distilled water and filter sterilized. Protein concentrations were determined using Bradford reagent (Bio-Rad Laboratories Inc., Hercules, CA). Molecular reagents were purchased from New England Biolabs, unless otherwise stated. Unless stated otherwise, all chemicals were purchased from Sigma-Aldrich (St. Louis, MO). DNA was sequenced at Azenta (South Plainfield, NJ).

### Plasmid and strain construction

Creating the transposon mutant library in the *cop*^-^ strain and determining the locations of the mutations was previous described [32]. Synthetic DNA (S4 Table) was synthesized by Twist Biosciences (San Francisco, CA) and DNA primers (S5 Table) were synthesized by Integrated DNA Technologies (Coralville, IA). Plasmids are listed in Table 5. All bacterial strains were PCR or sequenced verified before use.

Construction of the pSK_CRISPRi construct was done by combining two PCR amplicons and a digested plasmid using Gibson Assembly (NEB). The CRISPRi gene and sgRNA promoter and cloning site was based off the construct described here [43].The CRISPR product was synthesized in two pieces. The first fragment was amplified using the primers pSK Crispr fwd and mid rev 2 gibson. The second fragment was amplified using pSK Crispr rev and Crispr mid fwd. The pSK vector backbone carries a *Sap*I site that was removed by cloning the CRISPRi PCR into the *Sap*I site, the primers used to amplify the CRISPRi construct have overlapping sequences to the linearized vector. The CRISPR sequence was verified by sequencing using verify sgRNA fwd, verify Cas fwd, tetR verify fwd, verify dcas9 2 fwd, verify dcas9 3 fwd, and verify dcas9 4 fwd, which flanked the 6 kb sequence.

The pEPSA5 vectors were constructed by digesting the plasmid and respective amplicons with the restriction enzymes *Kpn*I and *Bam*HI or *Eco*RI and *Bam*HI and joined using the Quick Ligation™ Kit (NEB). A RBS was added to the 5’ end of the cloned gene via the primer. The pLL39_pglS vector was made by amplifying the pglS (SAUSA300_1902) using the following primer pair: pLL39-1902 *Pst*I rev and pLL39 *XbaI* 1902 fwd. The insert and vector were digested with XbaI and PstI before ligating.

The pJB38_*apt*::*tetM* plasmid was created using yeast homologous recombination cloning (YCC) in *Saccharomyces cerevisiae* FY2 as previously described [70, 71]. pJB38 was linearized with *Mlu*I and *Nhe*I and combined with DNA amplicons generated using the following primer pairs: YCCaptfor and apttetRrev; apttetRfor and tetRaptrev; pJB38aptrev and tetRaptfor. Chromosomal DNA from *srrAB*::*tetM* strain was used as a template for the PCR reactions [70]. The *apt*::*tetM* strain was created as previously described [66].

To create the *prs* expression vector for purification of Prs we generate an amplicon using the Prs5BamHI and Prs3SalI primer pair. The amplicon and pGEX-6P-1 (GE Healthcare) was digesting with *Bam*HI and *Sal*I. The agarose gel purified products were ligated using Quick Ligase (NEB) and transformed into *E*. *coli* DH5α. After sequence verification, the vector was then transformed into *E*. *coli* BL21 for *prs* expression.

### Whole cell metal quantification

*S*. *aureus* were grown and analyzed as described previously [31]. Briefly, cells were grown for 18 hours overnight in TSB before diluting them to an OD of 0.05 (A_600_) in 7.5 mL of Chelex (Bio-Rad)-treated TSB in a 30 mL capacity culture tubes. Cells were allowed to grow with shaking for eight hours, before 0 or 5 μM CuSO_4_ was added. Cultures were further incubated for 60 minutes. Pre-weighted metal-free 15 mL propylene tubes were used to pellet the cells using a prechilled tabletop centrifuge (Eppendorf, Hauppauge, NY). Pellets were washed three times with 10 mL of ice-cold PBS. All samples were kept at -80°C or on dry ice until processing. Cell pellets were acid digested and quantification was performed using an Agilent 7700 inductively coupled plasma mass spectrometer (Agilent, Santa Clara, CA). Data were acquired and analyzed using the Agilent Mass Hunter Workstation Software version A.01.02.

### RNA extraction, cDNA synthesis and qPCR

To analyze RNA abundances corresponding to genes targeted by CRISPRi, the *cop*^*-*^ strain carrying pSK_CRISPi vectors were cultures overnight and diluted into 5 mL of fresh TSB-Cm to OD_600_ 0.1 in 30 mL culture tubes. The cells cultured until an OD_600_ of 0.5 before the addition of 0 or 100 ng mL^-1^ anhydrotetracycline (Atet). After an additional one-hour culture, five mL of cells were harvested by centrifugation, washed with PBS, and resuspended in 500 μL with RNA protect (QIAGEN). RNA extraction, cDNA synthesis, and transcript quantification (QuantStudio 3, Bio-Rad Laboratories Inc., Hercules, CA) were performed as previously described [31].

## Metabolomic analyses and PRPP measurements

*Metabolomics*. All metabolic analyses were conducted using biological triplicates. Overnight cultures (*cop*^*-*^ or *cop- apt*::*tet*) were diluted into fresh TSB to OD_600_ 0.1, grown until OD_600_ of 0.5 in a flask. At this point, 5 mL of culture was transferred to individual 30 mL capacity culture tubes and exposed to 0 or 5 μM Cu(II) for three hours. Samples for the metabolite profiling were prepared described previously [72]. Briefly, 1 mL of the cells were pelleted, washed once with PBS, and resuspended in 1 mL of Methanol:Acetonitrile:Water (2:2:1) solution. Cells were lysed by bead beating (2 cycles, 40 s each, 6.0 m s-1) using a FastPrep homogenizer (MP Biomedicals) and 0.1-mm silica glass beads (MP Biomedicals). Samples were centrifuged twice at 14,500 rpm at 4°C for 2 minutes and supernatant retained. The supernatant was filtered with nylon membrane syringe filters (13 mm, 0.22 μm, Fisherbrand) and samples were store at -80°C until metabolite analysis was performed.

Samples were analyzed at the metabolomics core of the Cancer Institute of New Jersey. HILIC separation was performed on a Vanquish Horizon UHPLC system (Thermo Fisher Scientific, Waltham, MA) with an XBridge BEH Amide column (150 mm × 2.1 mm, 2.5 μm particle size, Waters, Milford, MA) using a gradient of solvent A (95%:5% H_2_O:acetonitrile with 20 mM acetic acid, 40 mM ammonium hydroxide, pH 9.4) and solvent B (20%:80% H_2_O:acetonitrile with 20 mM acetic acid, 40 mM ammonium hydroxide, pH 9.4). The gradient was 0 min, 100% B; 3 min, 100% B; 3.2 min, 90% B; 6.2 min, 90% B; 6.5 min, 80% B; 10.5 min, 80% B; 10.7 min, 70% B; 13.5 min, 70% B; 13.7 min, 45% B; 16 min, 45% B; 16.5 min, 100% B; and 22 min, 100% B. The flow rate was 300 μL min^-1^. The column temperature was set to 25°C. The autosampler temperature was set to 4°C, and the injection volume was 5 μL. MS scans were obtained in negative mode with a resolution of 70,000 at m/z 200, in addition to an automatic gain control target of 3 x 106 and m/z scan range of 72 to 1000. Metabolite data was obtained using the MAVEN software package [73] (mass accuracy window: 5 ppm). The data from these experiments are included as S1 and S3 Tables.

Carbon labeling experiments were performed using D-Glucose-^13^C_6_ (389374, Sigma-Aldrich) using the *cop*^*-*^ strain. Overnight cultures were diluted to an OD_600_ of 0.1 in 1.5 mL of fresh glucose-free TSB supplemented with 11 mM glucose in 10 mL capacity culture tube. Cells were cultured to an OD_600_ of 0.5 cells, pelleted, washed twice with PBS, and resuspended in 1.5 mL of glucose-free TSB supplemented with 11 mM ^13^C_6_ glucose. Cells were cultured for 15, 30 or 60 additional minutes before samples were collected and metabolites were extracted as described above. The data from these experiments is included as S2 Table.

*PRPP quantification*. The *cop*^*-*^ and *cop- apt*::*tetM* strains vector were grown O.N. in TSB and diluted to an OD_600_ of 0.1 in TSB medium. Cells were cultured until an OD_600_ of 0.5 and 0 or 5 μM Cu(II) was added. Cells were cultured for an additional three hours before collecting 0.5 mL of cells and washing them with PBS. Cells were resuspended in 1 mL of PBS and lysed by bead beating using 0.1-mm silica glass beads (MP Biomedicals) as previously described [74]. Cell debris was removed by centrifugation 4°C using a tabletop microcentrifuge. PRPP levels were measured using the PRPP ELISA Kit (Mybiosource) following manufacture instructions. PRPP levels were standardized to protein concentration which was determined using Bradford reagent.

### Protein purification and enzymatic assays

*Prs purification*. An overnight culture of *E*. *coli* BL21 carrying pGEX-6P-1_*prs* was sub-cultured to OD_600_ of 0.1 into LB-AMP medium. Cells were cultured for 1.5 hours at 37°C and 0.1 mM IPTG was added. Cells were cultured for four more hours before harvesting by centrifugation. Cell pellets were washed with PBS and cells were resuspended in lysis buffer (50 mM phosphate buffer pH 7.5, 100 mM NaCl, 0.01 mg mL^-1^ DNase and 1 mg mL^-1^ lysozyme). Cells were lysed by sonication (Vibra-Cell; Sonics, Newtown, CT) on ice for 20 min at 40% (1s on / 1s off pulse). Cell debris was removed by centrifugation and supernatant was filter through a 0.22 μm polypropylene filter before loading onto GSTrap 4B Columns (Millipore Sigma). Protein was purified according to manufactures instructions. Purified Prs was dialyzed five times in a 1:1000 ratio (protein:buffer) against dialysis buffer (50 mM phosphate buffer, 150 mM NaCl, pH 7.5) at 4°C before use.

*Aconitase activity assay*. Cells were prepared as previously described [31]. Aconitase activity was measure as previously described [70] using a using Cary 60 UV-Vis spectrophotometer (Agilent, Santa Clara, CA).

*Prs activity assays*. For *in vivo* assays, the *cop*^*-*^ strain carrying the pEPSA5_*prs* vector was cultured overnight in TSB-Cm. The was then diluted to an OD_600_ of 0.1 in 1.5 mL of TSB-Cm supplemented with 0.5% xylose. Cultures were grown as indicated for metabolomics, one mL of cells were collected by centrifugation, and washed twice with PBS. Cells were resuspended in 1 mL of PBS and lysed by bead beating using 0.1-mm silica glass beads (MP Biomedicals). Cell debris was removed by centrifugation 4°C using a tabletop microcentrifuge and supernatants were concentrated using Vivaspin 500 (3,000K MWCO, PES, Sartorius). Prs activity was measured by monitoring AMP formation using a coupled enzyme system [46]. For each extract, we subtracted the basal NADH oxidation activity (before addition R5P) from the R5P-dependent NADH oxidation. This was necessary due to the presence of many other enzymes that consume ATP and produce AMP. Measurements were carried out using UV-STAR microplates (Greiner Bio-One) and Varioskan LUX Multimode Microplate Reader (Thermo Scientific). *in vitro* Prs activity assays contained 0.07 mg mL^-1^ Prs. Cu(I) was prepared using asorbic acid as previously described [5].

### Murine infection assays

C57BL/6 mice, 6 weeks of age, were intranasally infected with 2–4 x 10^7^ cfu of *S*. *aureus* in 50 μL of PBS under anesthesia (ketamine and xylazine). Bacterial loads were enumerated at 24 h post infection from bronchoalveolar lavage fluid (BALF) by washing the airway 3 times with 1 ml of PBS and homogenized lung tissue. Skin infection was initiated with 2–4 x 10^6^ cfu of *S*. *aureus* in 100 μL of PBS delivered subcutaneously. Flow cytometry was performed on fluorescently labelled cell from BALF as described previously [75]. Bacterial counts were quantified by using CHROMagar *S*. *aureus* plates (BD Biosciences).

### Statistical analysis

One-way ANOVA followed by a Dunnet test analysis was performed for multiple group comparison to a control. For two group comparisons (controls vs treatment or between bacterial strains), student’s t-tests were performed. Multiple group comparisons for animal data were performed using an ANOVA with a Kruskal-Wallis test or a Mann-Whitney non-parametric test for two group comparisons. All analyses were conducted with Sigmaplot 11, Microsoft, Excel, or Prism 9.

## Supporting information

S1 Fig Panels A and B; the number of ^13^C atoms in individual NAD^+^ or NADH molecules after growth of the *cop*^-^ strain in TSB medium containing ^13^C glucose. Data represent the average of three biological replicates and standard deviations are displayed. Panel C; the number of ^13^C atoms incorporated into individual molecules of nicotinamide dinucleotide (NADH + NAD^+^) after growth in TSB medium containing ^13^C glucose.
(TIFF)

S2 Fig The optical densities of cultures of the *cop*^-^ and *cop*^-^ *pglS*::*Tn* strains were recorded after 18 hours of static growth in TSB media with 0–1 mM Cu(II) are presented. The data presented represent the average of three biological replicates and standard deviations are displayed; however, they are too small to be seen for most data points. Student’s t-tests were performed between culture optical density readings at each individual Cu(II) concentration and * indicates p < 0.05.
(TIFF)

S3 Fig Growth the *cop*^-^ and *cop*^*-*^ *apt*::*tetM* strains in liquid chemically defined media with and without 30 μM Cu(II). The data represent the average of three biological triplicates with standard deviations shown. Note that in some cases the errors bars are smaller than the data points.
(TIFF)

S4 Fig The *cop*^-^ and *cop*^*-*^ *apt*::*tetM* strain accumulate similar Cu loads. The *cop*^-^ and *cop*^*-*^ *apt*::*tetM* strains were cultured in TSB media with and without 5 μM Cu(II) before cells were harvested and total cell associated metal was determined by ICP-MS. Data represent the average of three biological replicates and standard deviations are displayed. Student’s t-tests were performed on the data and * indicates p < 0.05.
(TIFF)

S5 Fig The *cop*^-^ and *cop*^*-*^ *apt*::*tetM* strains have similar aconitase (AcnA) activity after growth with Cu(II). The *cop*^-^ and *cop*^*-*^ *apt*::*tetM* strains were cultured with and without 5 μM Cu(II) before the activity was monitored in cell-free lysates. Data represent the average of three biological replicates and standard deviations are displayed. Student’s t-tests were performed on the data and * indicates p < 0.05.
(TIFF)

S6 Fig Growth of the *cop*^-^ strain containing pEPSA5, pEPSA5_*apt*, or pEPSA5_*prs* in TSB-Cm media with and without Cu(II). Overnight cultures in TSB-Cm were back diluted to an optical density of 0.001 in media containing Cm and 1% xylose Cu(II) was added at the indicated concentrations. Culture optical densities were measured after 18 hours of growth. Data represented the average of biological triplicates with standard deviations shown. Student’s t-tests were performed on the data and * indicates p < 0.05.
(TIFF)

S7 Fig Genetically altering the demand for PRPP modulates the sensitivity to Cu(II). Cultures of the *cop*^*-*^ strain with a plasmid were serial diluted and strains were spot plated on defined media with chloramphenicol and 0 or 1.4 μM Cu(II). Panel A, the *cop*^-^ strain containing pEPSA5 or pEPSA5_*purE* were spot plated with or without 50 μM hypoxanthine or orotate. Panel B, the *cop*^-^ strain containing pEPSA5 or pEPSA5_*htp* were spot plated with or without 50 μM hypoxanthine. Panel C, the *cop*^-^ strain containing pEPSA5 or pEPSA5_*1804* were spot plated with or without 50 μM nicotinate. Panel D, the *cop*^-^ strain containing pEPSA5 or pEPSA5_*upp* were spot plated with or without 50 μM uridine. Panel E, the *cop*^-^ strain containing pEPSA5 or pEPSA5_*apt* were spot plated with or without 50 μM adenine. Photos of representative experiments displayed. The dilutions displayed are from 10^−2^ to 10^−6^.
(TIFF)

S8 Fig Growth the *cop*^-^ strain in liquid chemically defined media with and without 8 μM Cu(II). The complimented media was supplemented with 50 μM of uridine, tryptophan, guanosine, and nicotinamide mononucleotide (NMN). The data represent the average of three biological triplicates with standard deviations shown. Note that in some cases the errors bars are smaller than the data points. Student’s t-tests were performed on the Cu(II) treated samples with without chemical complementation data and * indicates p < 0.05 for the time point indicated.
(TIFF)

S9 Fig Decreased transcription of *prs* results in decreased growth. Cultures of the *cop*^-^ strain containing a pSK_CRISPRi vector were serial diluted and spot plated on TSA chloramphenicol medium with or without 100 ng mL^-1^ anhydrotetracycline (Atet). The gene targeted by the sgRNA are displayed except for the control which contains a randomized sgRNA (pSK_CRIPSRi).
(TIFF)

S10 Fig The pSK_CRISPRi_*hla* vector can decrease *hla* expression when induced. Panel A, two μL of the *cop*^-^ strain containing a pSK_CRISPRi vector were serial diluted and spot plated on TSA chloramphenicol blood agar media with or without 100 ng mL^-1^ anhydrotetracycline (Atet). The gene targeted by the sgRNA are displayed except for the control which contains a randomized sgRNA (pSK_CRIPSRi). Panel B; the abundances of RNAs corresponding to *hla* were quantified from the same strains listed in Panel A after culture in TSB chloramphenicol medium with (light bars) and without (dark bars) 100 ng mL^-1^ Atet.
(TIFF)

S11 Fig The enzymes utilized to aid in monitoring Prs activity are not inhibited by Cu(I) *in vitro*. The concentration of the coupling enzymes was the same as used to monitor Prs activity, but Prs was not included. The reaction was initiated by the addition 10mM AMP and NADH oxidation was monitored.
(TIFF)

S1 Table Metabolite concentrations in the *cop*^-^ strain after 30, 60 and 90 minutes of adding 0 or 5 μM Cu(II). Experiment was conducted using biological triplicates and ion counts were standardized to culture optical density (A_600_).
(XLSX)

S2 Table Concentrations of ^13^C carbon atoms in select metabolites after growth in TSB medium containing ^13^C glucose. Experiment was conducted using biological triplicates of the *cop*^-^ strain and ion counts were standardized to culture optical density (A_600_).
(XLSX)

S3 Table Metabolite concentrations in the *cop*^-^ and *cop*^-^ *apt*::*tetM* strains. Experiment was conducted using biological triplicates and ion counts were standardized to culture optical density (A_600_).
(XLSX)

S4 Table DNA fragments synthesized to create the pSK_CRISPRi transcription knockdown vector. (DOCX)

S5 Table DNA primers used in this study. (DOCX)
