# Supplementary figures and images for: Copper ions inhibit pentose phosphate pathway function in Staphylococcus aureus
Source: PLoS Pathog. 2023 May 26;19(5):e1011393. doi: 10.1371/journal.ppat.1011393 (PMC10249872; doi:10.1371/journal.ppat.1011393)

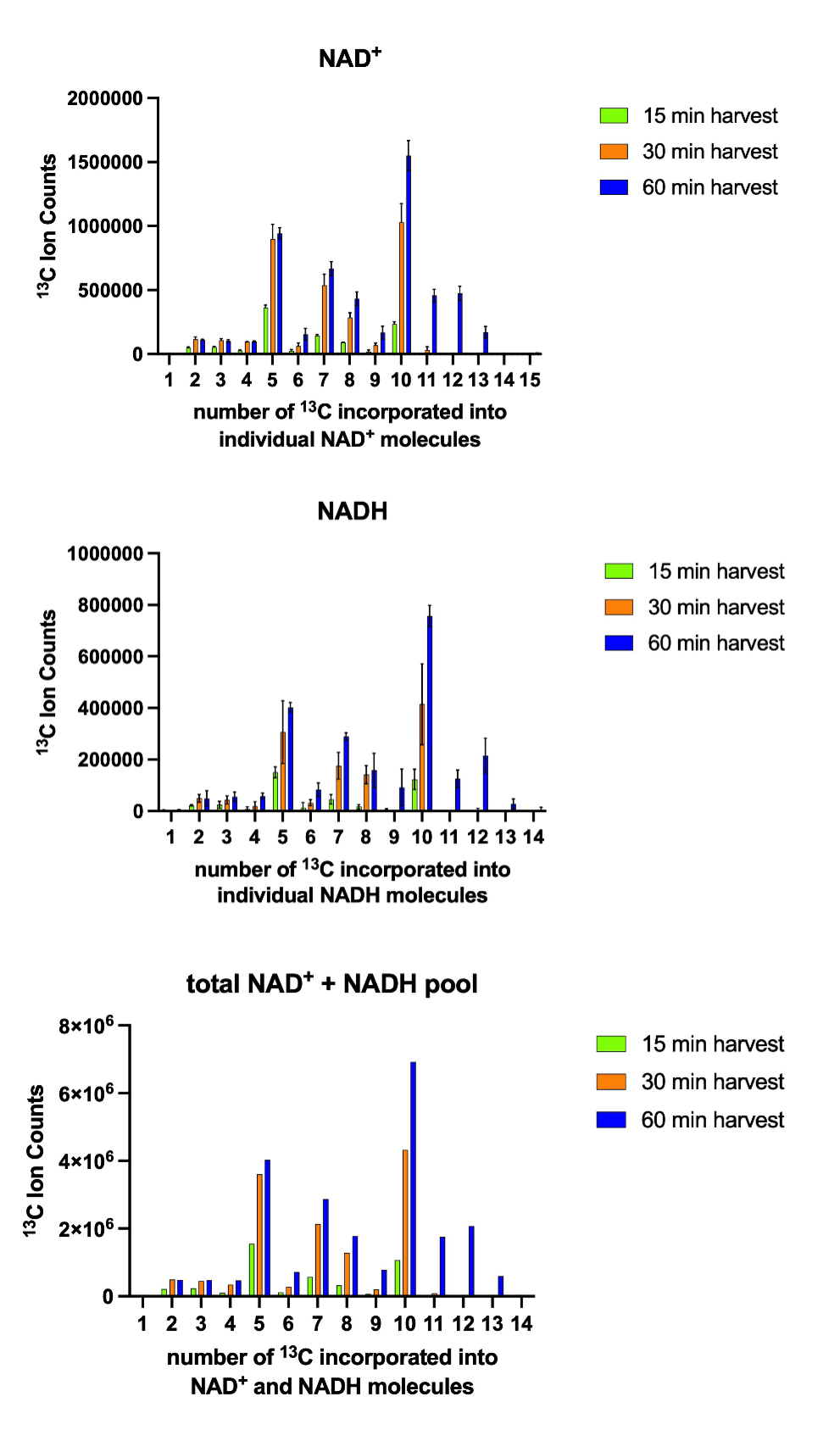

Supplement: S1 Fig — Data represent the average of three biological replicates and standard deviations are displayed. Panel C; the number of 13C atoms incorporated into individual molecules of nicotinamide dinucleotide (NADH + NAD+) after growth in TSB medium containing 13C glucose. (TIFF) [file ppat.1011393.s001.tiff]

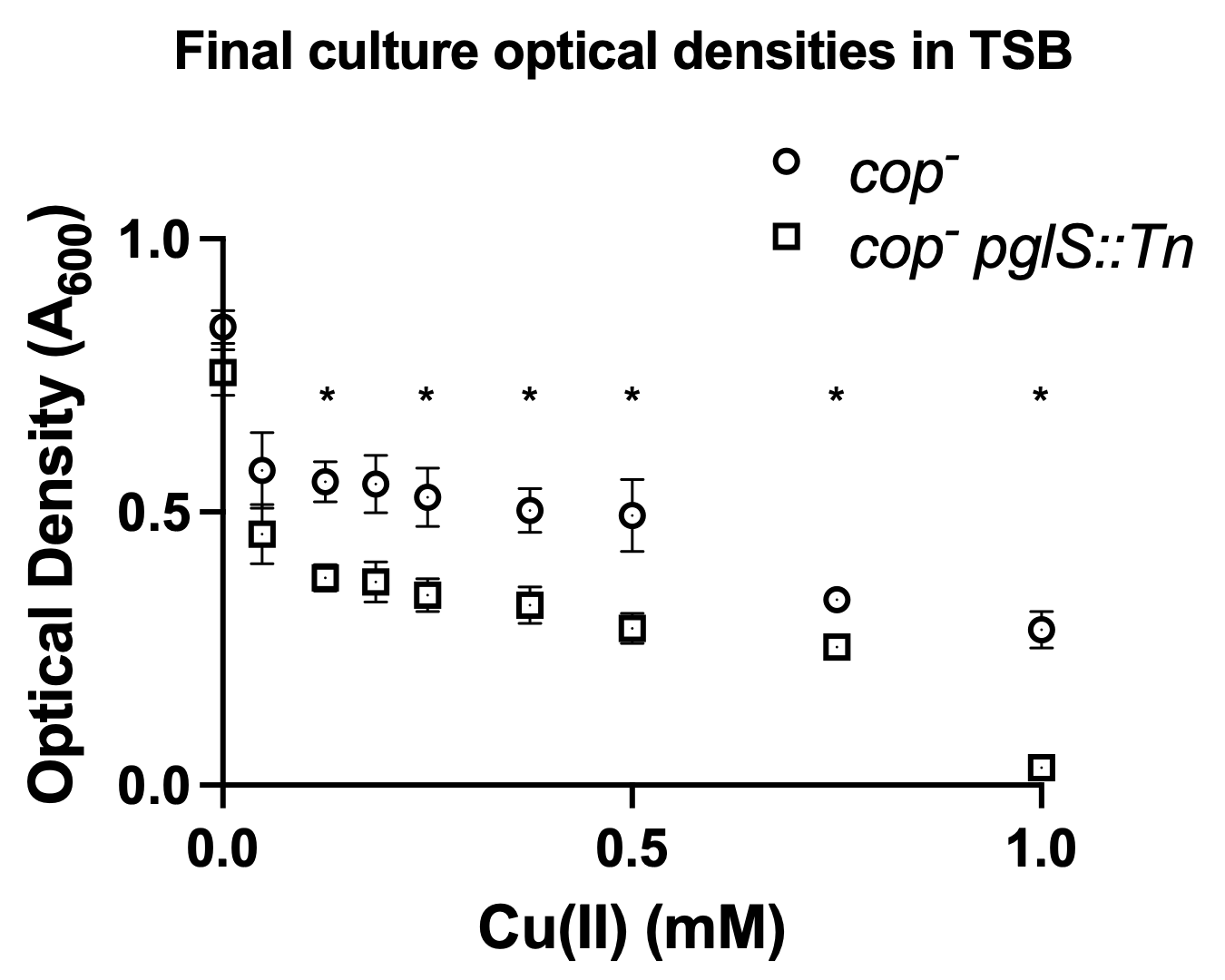

Supplement: S2 Fig — The data presented represent the average of three biological replicates and standard deviations are displayed; however, they are too small to be seen for most data points. Student’s t-tests were performed between culture optical density readings at each individual Cu(II) concentration and * indicates p < 0.05. (TIFF) [file ppat.1011393.s002.tiff]

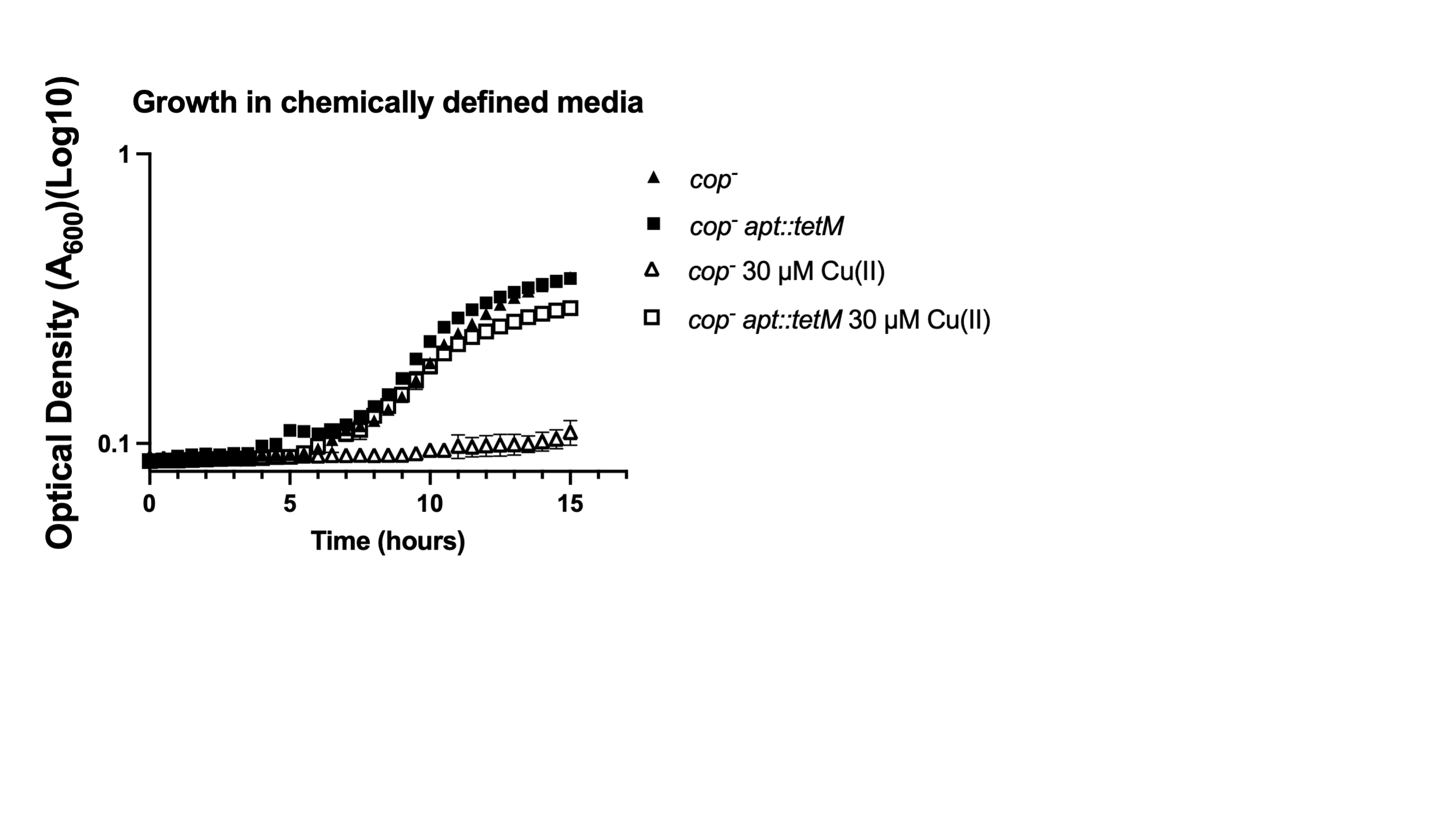

Supplement: S3 Fig — The data represent the average of three biological triplicates with standard deviations shown. Note that in some cases the errors bars are smaller than the data points. (TIFF) [file ppat.1011393.s003.tiff]

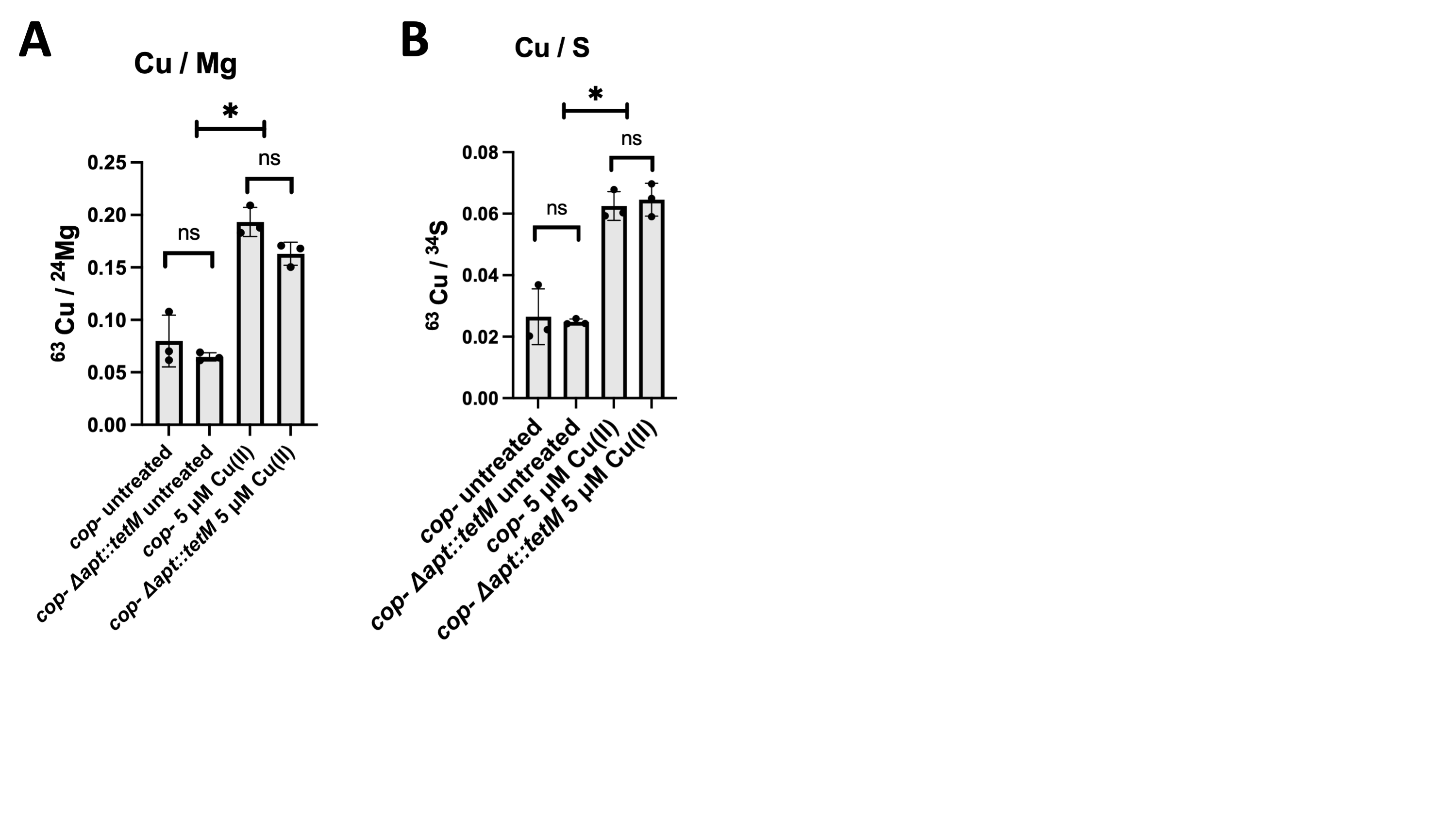

Supplement: S4 Fig — The cop- and cop- apt::tetM strains were cultured in TSB media with and without 5 μM Cu(II) before cells were harvested and total cell associated metal was determined by ICP-MS. Data represent the average of three biological replicates and standard deviations are displayed. Student’s t-tests were performed on the data and * indicates p < 0.05. (TIFF) [file ppat.1011393.s004.tiff]

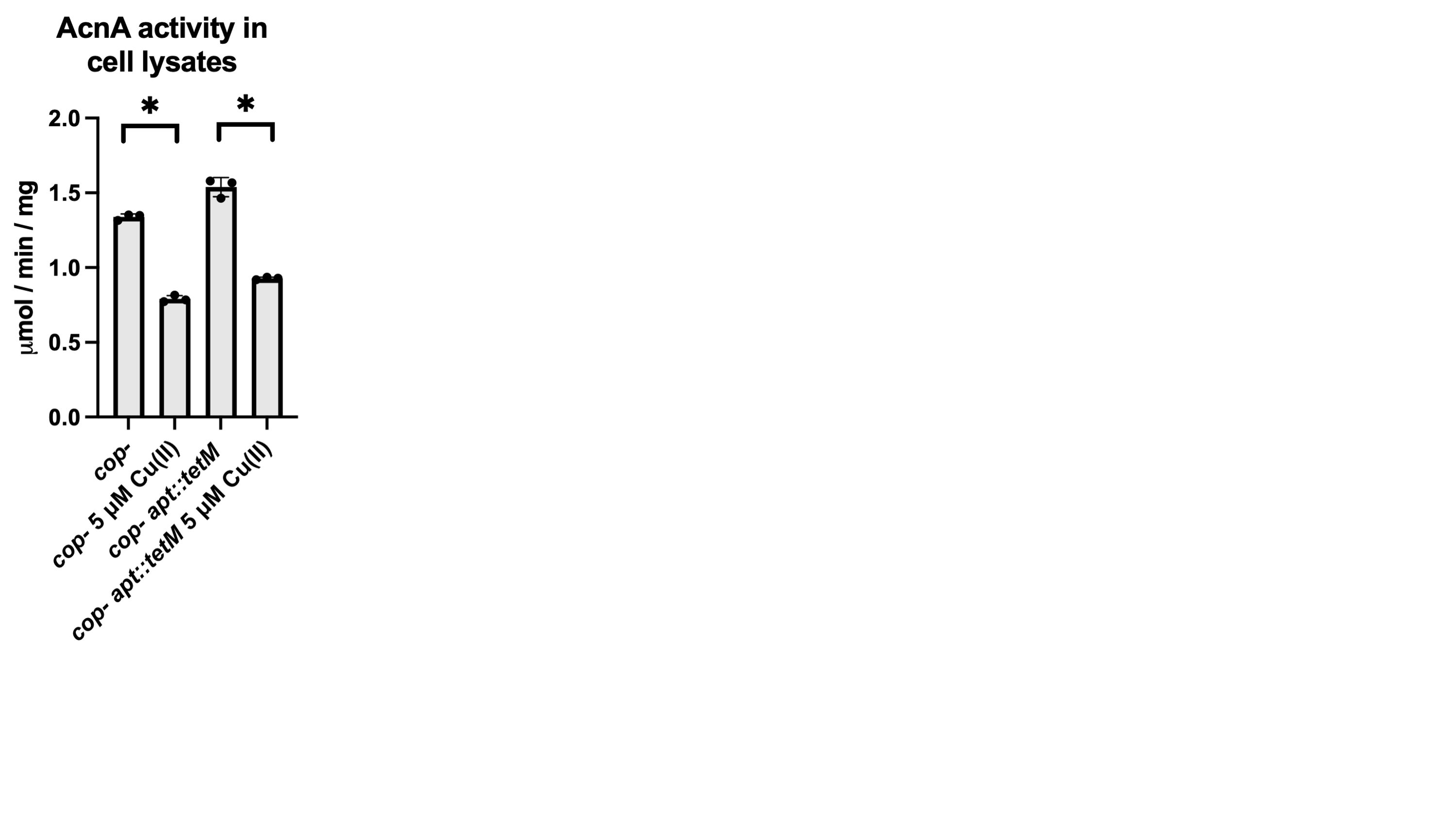

Supplement: S5 Fig — The cop- and cop- apt::tetM strains were cultured with and without 5 μM Cu(II) before the activity was monitored in cell-free lysates. Data represent the average of three biological replicates and standard deviations are displayed. Student’s t-tests were performed on the data and * indicates p < 0.05. (TIFF) [file ppat.1011393.s005.tiff]

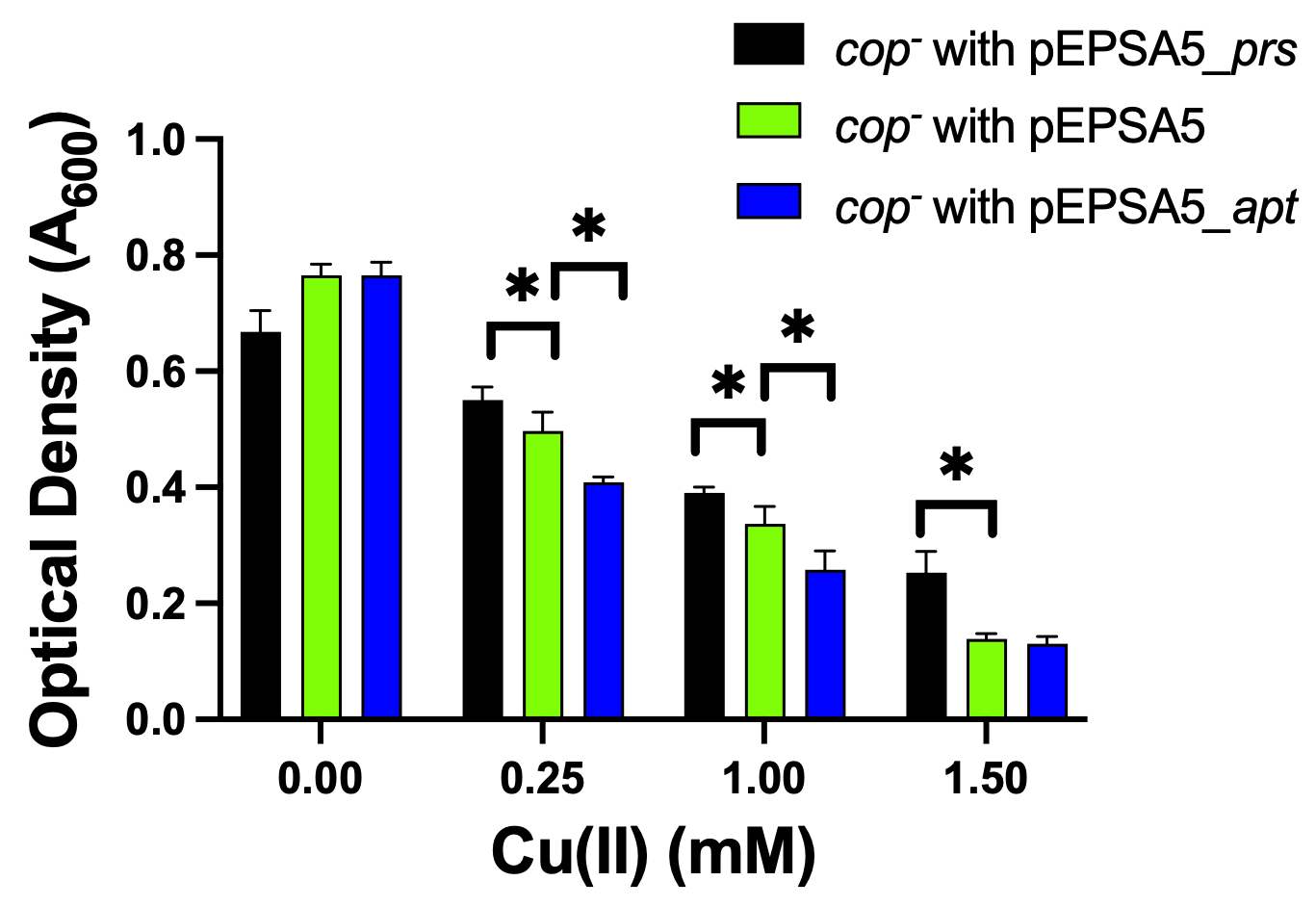

Supplement: S6 Fig — Overnight cultures in TSB-Cm were back diluted to an optical density of 0.001 in media containing Cm and 1% xylose Cu(II) was added at the indicated concentrations. Culture optical densities were measured after 18 hours of growth. Data represented the average of biological triplicates with standard deviations shown. Student’s t-tests were performed on the data and * indicates p < 0.05. (TIFF) [file ppat.1011393.s006.tiff]

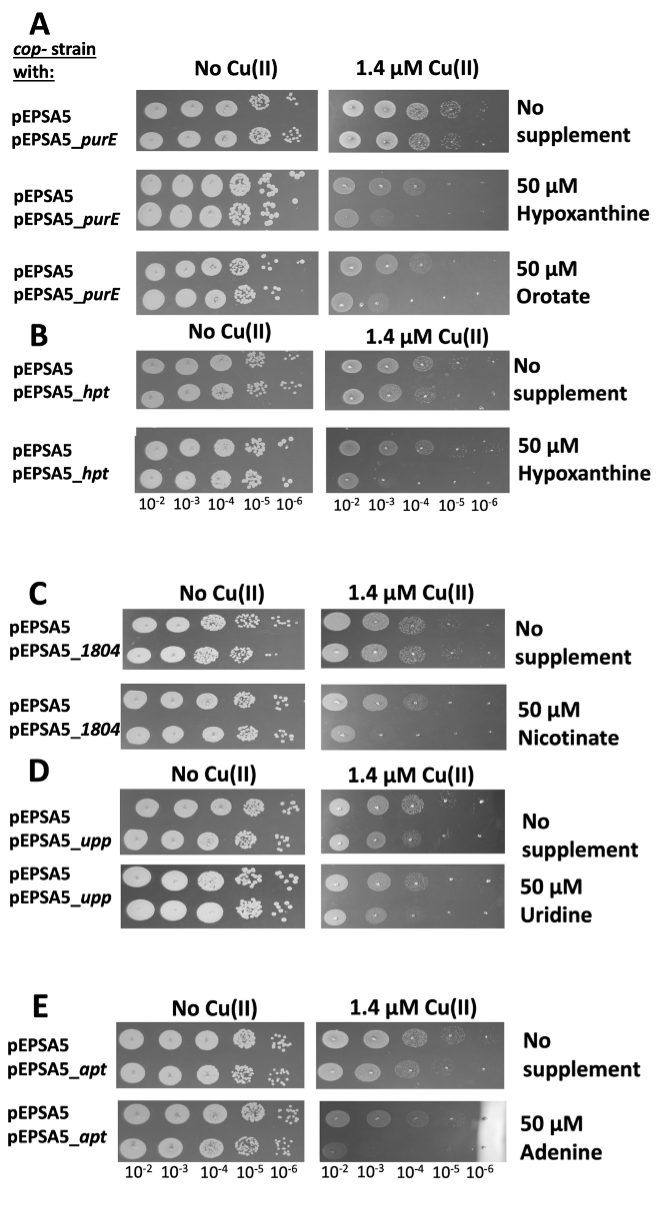

Supplement: S7 Fig — Cultures of the cop- strain with a plasmid were serial diluted and strains were spot plated on defined media with chloramphenicol and 0 or 1.4 μM Cu(II). Panel A, the cop- strain containing pEPSA5 or pEPSA5_purE were spot plated with or without 50 μM hypoxanthine or orotate. Panel B, the cop- strain containing pEPSA5 or pEPSA5_htp were spot plated with or without 50 μM hypoxanthine. Panel C, the cop- strain containing pEPSA5 or pEPSA5_1804 were spot plated with or without 50 μM nicotinate. Panel D, the cop- strain containing pEPSA5 or pEPSA5_upp were spot plated with or without 50 μM uridine. Panel E, the cop- strain containing pEPSA5 or pEPSA5_apt were spot plated with or without 50 μM adenine. Photos of representative experiments displayed. The dilutions displayed are from 10−2 to 10−6. (TIFF) [file ppat.1011393.s007.tiff]

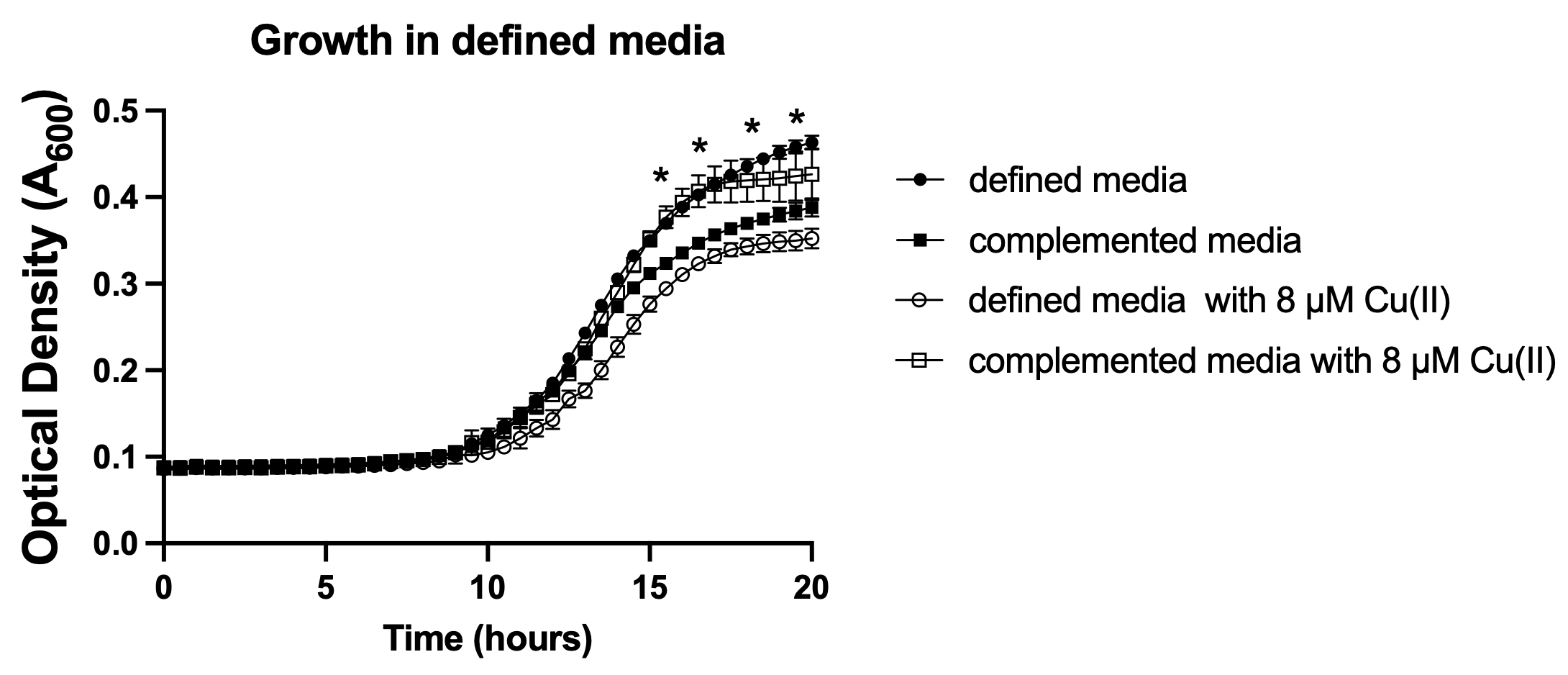

Supplement: S8 Fig — The complimented media was supplemented with 50 μM of uridine, tryptophan, guanosine, and nicotinamide mononucleotide (NMN). The data represent the average of three biological triplicates with standard deviations shown. Note that in some cases the errors bars are smaller than the data points. Student’s t-tests were performed on the Cu(II) treated samples with without chemical complementation data and * indicates p < 0.05 for the time point indicated. (TIFF) [file ppat.1011393.s008.tiff]

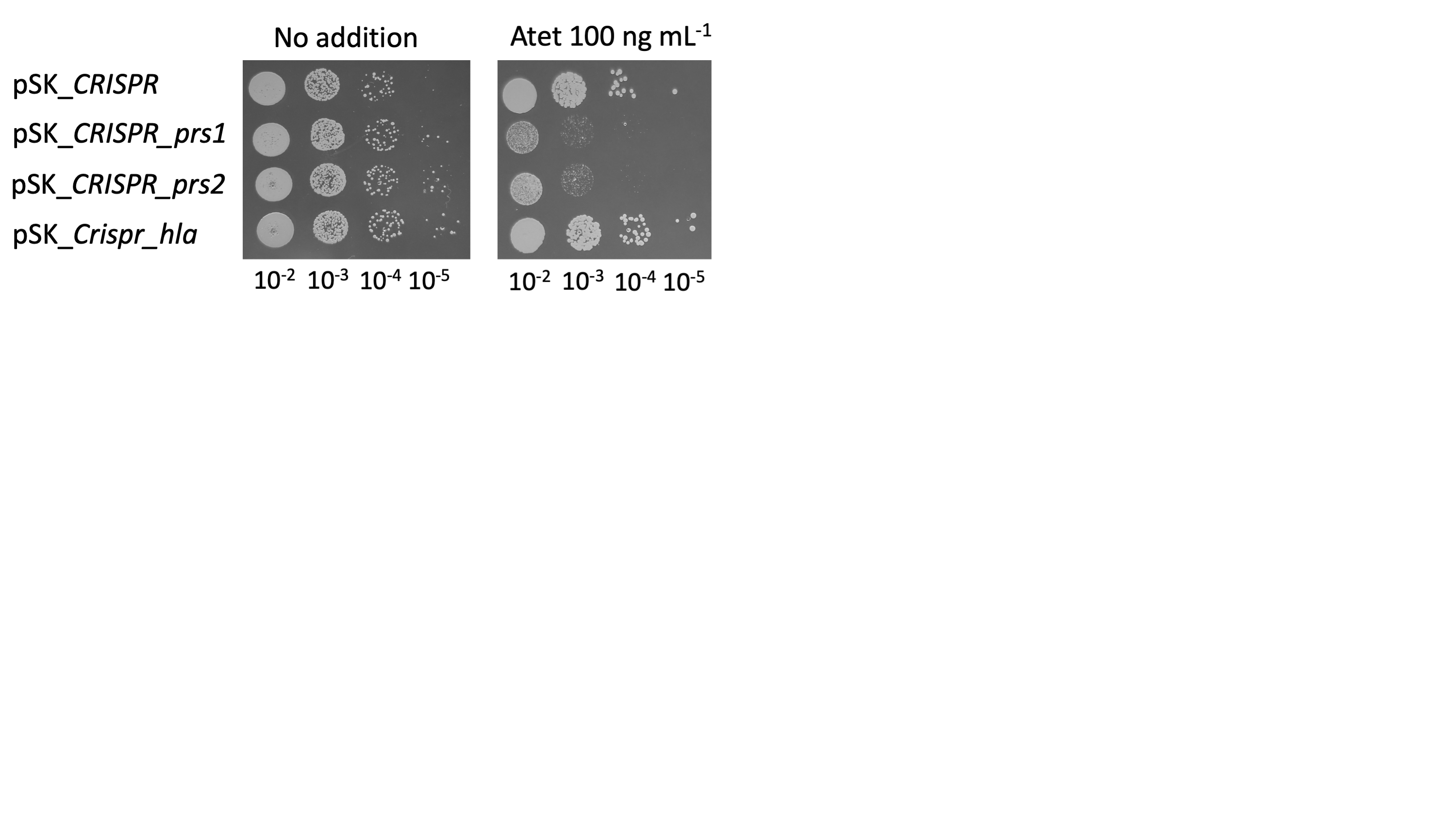

Supplement: S9 Fig — Cultures of the cop- strain containing a pSK_CRISPRi vector were serial diluted and spot plated on TSA chloramphenicol medium with or without 100 ng mL-1 anhydrotetracycline (Atet). The gene targeted by the sgRNA are displayed except for the control which contains a randomized sgRNA (pSK_CRIPSRi). (TIFF) [file ppat.1011393.s009.tiff]

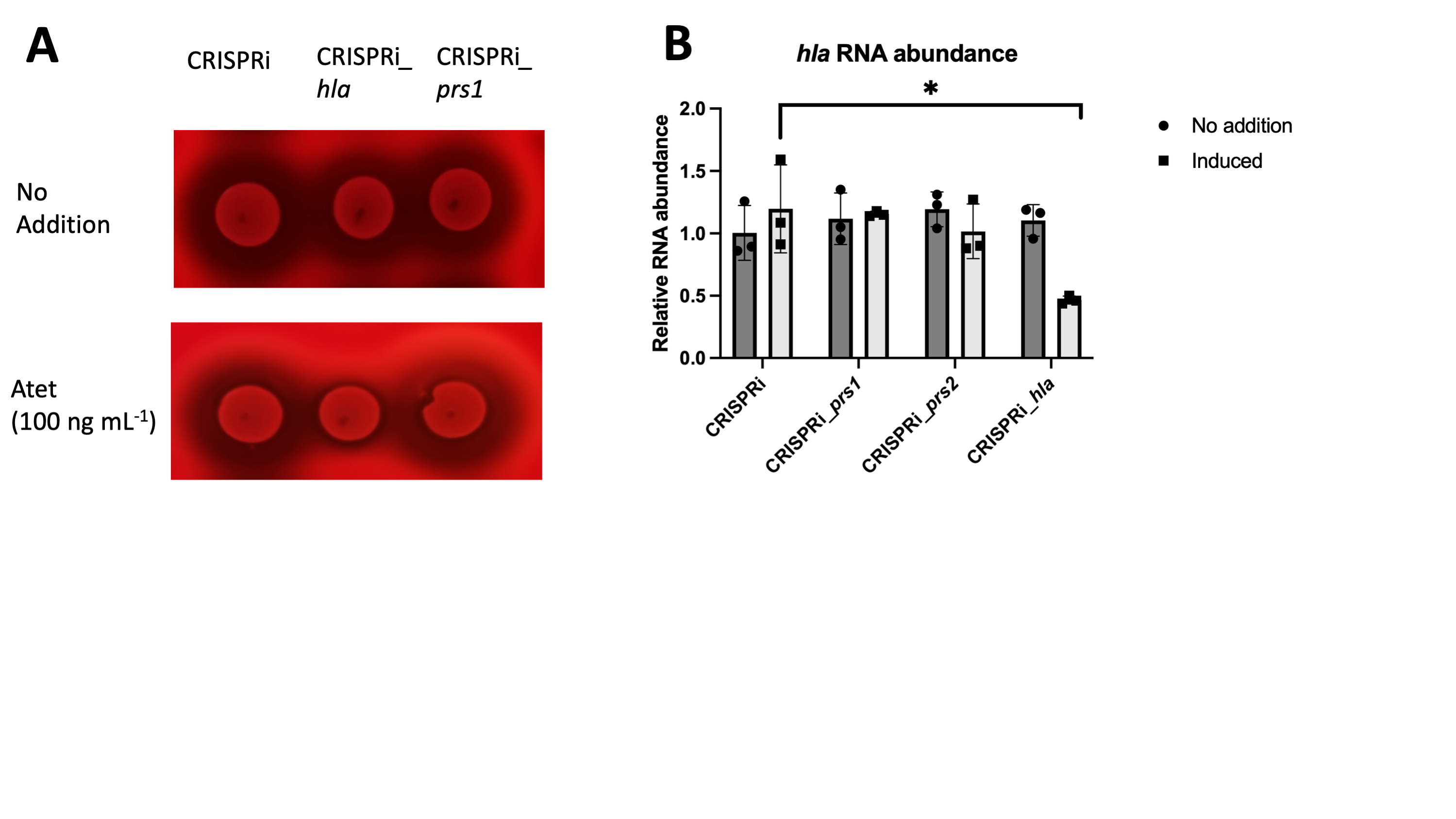

Supplement: S10 Fig — Panel A, two μL of the cop- strain containing a pSK_CRISPRi vector were serial diluted and spot plated on TSA chloramphenicol blood agar media with or without 100 ng mL-1 anhydrotetracycline (Atet). The gene targeted by the sgRNA are displayed except for the control which contains a randomized sgRNA (pSK_CRIPSRi). Panel B; the abundances of RNAs corresponding to hla were quantified from the same strains listed in Panel A after culture in TSB chloramphenicol medium with (light bars) and without (dark bars) 100 ng mL-1 Atet. (TIFF) [file ppat.1011393.s010.tiff]

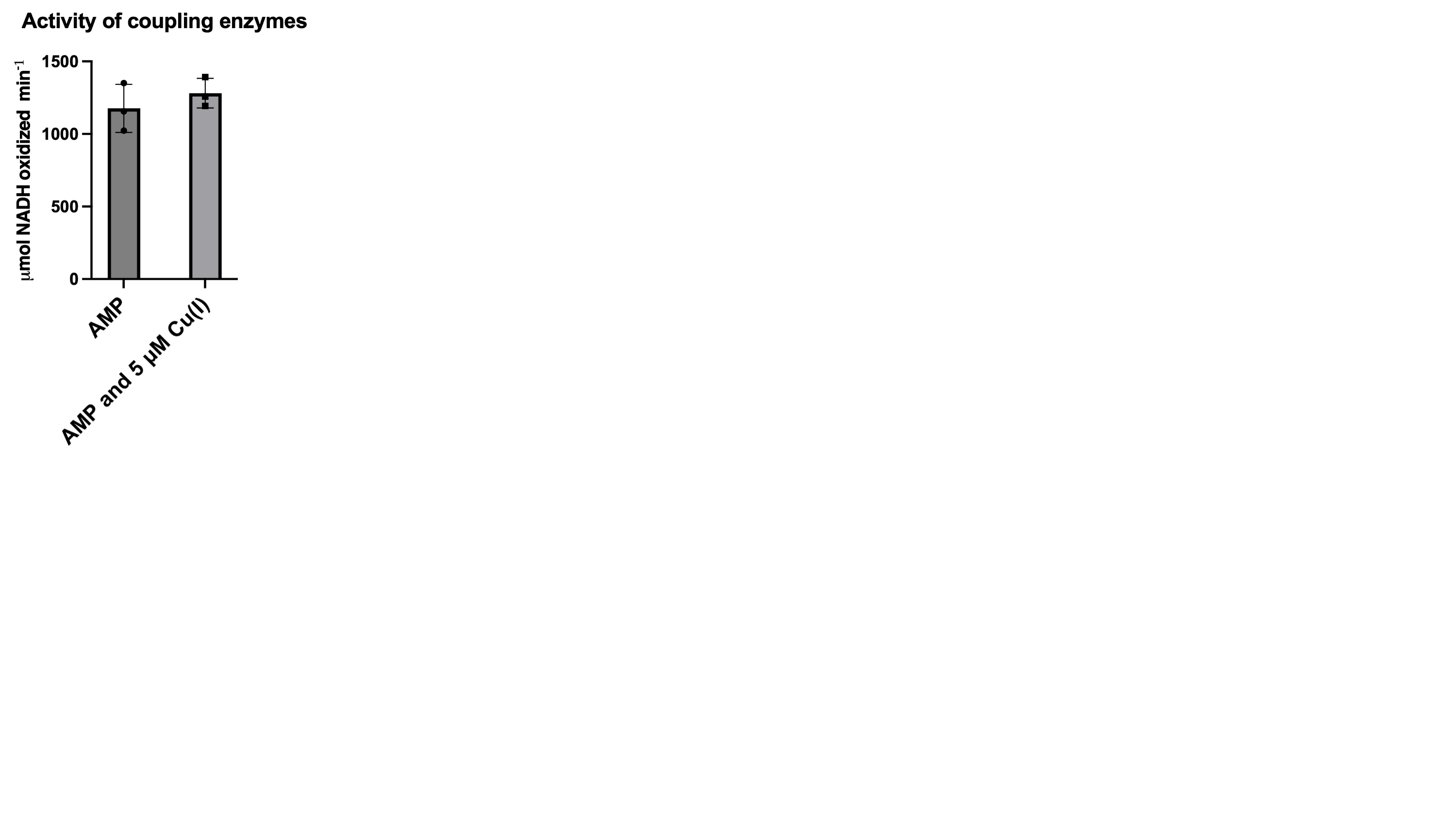

Supplement: S11 Fig — The concentration of the coupling enzymes was the same as used to monitor Prs activity, but Prs was not included. The reaction was initiated by the addition 10mM AMP and NADH oxidation was monitored. (TIFF) [file ppat.1011393.s011.tiff]
