# Supplementary material for: Copper ions inhibit pentose phosphate pathway function in Staphylococcus aureus
Source: PLoS Pathog. 2023 May 26;19(5):e1011393. doi: 10.1371/journal.ppat.1011393 (PMC10249872; doi:10.1371/journal.ppat.1011393)
Supplement: S4 Table — (DOCX) [file ppat.1011393.s015.docx]

**S4 Table. DNA fragments synthesized.**

Synthesis fragment one

GAATTCAAAAAAGCACCGACTCGGTGCCACTTTTTCAAGTTGATAACGGACTAGCCTTATTTTAACTTGCTATTTCTAGCTCTAAAACAGAAGAGCCTGACATGGCTCTTCATAGTCATTGTAGCATGTTTGTTGTGATTTTTTTCACATAAATTATGTACAATTTTCATCAAATGTATGTCAAAACATAGAAACTGTACTAATTTGTGTAACAGTAATATGCTAATTGCATTCTGTTACACAACAGTTATAATGTTCACTATTTTCATTGCTTTAAAAGTTAATTTAGGAGTATATCCCGGGTATAAACGCAGAAAGGCCCACCCGAAGGTGAGCCAGTGTGACTCTAGTAGAGAGCGTTCACCGACAAACAACAGATAAAACGAAAGGCCCAGTCTTTCGACTGAGCCTTTCGTTTTATTTGATGCCTGGCTCTAGTTCAGTCACCTCCTAGCTGACTCAAATCAATGCGTGTTTCATAAAGACCAGTGATGGATTGATGGATAAGAGTGGCATCTAAAACTTCTTTTGTAGACGTATATCGTTTACGATCAATTGTTGTATCAAAATATTTAAAAGCAGCGGGAGCTCCAAGATTCGTCAACGTAAATAAATGAATAATATTTTCTGCTTGTTCACGTATTGGTTTGTCTCTATGTTTGTTATATGCACTAAGAACTTTATCTAAATTGGCATCTGCTAAAATAACACGCTTAGAAAATTCACTGATTTGCTCAATAATCTCATCTAAATAATGCTTATGCTGCTCCACAAACAATTGTTTTTGTTCGTTATCTTCTGGACTACCCTTCAACTTTTCATAATGACTAGCTAAATATAAAAAATTCACATATTTGCTTGGCAGAGCCAGCTCATTTCCTTTTTGTAATTCTCCGGCACTAGCCAGCATCCGTTTACGACCGTTTTCTAACTCAAAAAGACTATATTTAGGTAGTTTAATGATTAAGTCTTTTTTAACTTCCTTATATCCTTTAGCTTCTAAAAAGTCAATCGGATTTTTTTCAAAGGAACTTCTTTCCATAATTGTGATCCCTAGTAACTCTTTAACGGATTTTAACTTCTTCGATTTCCCTTTTTCCACCTTAGCAACCACTAGGACTGAATAAGCTACCGTTGGACTATCAAAACCACCATATTTTTTTGGATCCCAGTCTTTTTTACGAGCAATAAGCTTGTCCGAATTTCTTTTTGGTAAAATTGACTCCTTGGAGAATCCGCCTGTCTGTACTTCTGTTTTCTTGACAATATTGACTTGGGGCATGGACAATACTTTGCGCACTGTGGCAAAATCTCGCCCTTTATCCCAGACAATTTCTCCAGTTTCCCCATTAGTTTCGATTAGAGGGCGTTTGCGAATCTCTCCATTTGCAAGTGTAATTTCTGTTTTGAAGAAGTTCATGATATTAGAGTAAAAGAAATATTTTGCGGTTGCTTTGCCTATTTCTTGCTCAGACTTAGCAATCATTTTACGAACATCATAAACTTTATAATCACCATAGACAAACTCCGATTCAAGTTTTGGATATTTCTTAATCAAAGCAGTTCCAACGACGGCATTTAGATACGCATCATGGGCATGATGGTAATTGTTAATCTCACGTACTTTATAGAATTGGAAATCTTTTCGGAAGTCAGAAACTAATTTAGATTTTAAGGTAATCACTTTAACCTCTCGAATAAGTTTATCATTTTCATCGTATTTAGTATTCATGCGACTATCCAAAATTTGTGCCACATGCTTAGTGATTTGGCGAGTTTCAACCAATTGGCGTTTGATAAAACCAGCTTTATCAAGTTCACTCAAACCTCCACGTTCAGCTTTCGTTAAATTATCAAACTTACGTTGAGTGATTAACTTGGCGTTTAGAAGTTGTCTCCAATAGTTTTTCATCTTTTTGACTACTTCTTCACTTGGAACGTTATCCGATTTACCACGATTTTTATCAGAACGCGTTAAGACCTTATTGTCTATTGAATCGTCTTTAAGGAAACTTTGTGGAACAATGGCATCGACATCATAATCACTTAAACGATTAATATCTAATTCTTGGTCCACATACATGTCTCTTCCATTTTGGAGATAATAGAGATAGAGCTTTTCATTTTGCAATTGAGTATTTTCAACAGGATGCTCTTTAAGAATCTGACTTCCTAATTCTTTGATACCTTCTTCGATTCGTTTCATACGCTCTCGCGAATTTTTCTGGCCCTTTTGAGTTGTCTGATTTTCACGTGCCATTTCAATAACGATATTTTCTGGCTTATGCCGCCCCATTACTTTGACCAATTCATCAACAACTTTTACAGTCTGTAAAATACCTTTTTTAATAGCAGGGCTACCAGCTAAATTTGCAATATGTTCATGTAAACTATCGCCTTGTCCAGACACTTGTGCTTTTTGAATGTCTTCTTTAAATGTCAAACTATCATCATGGATCAGCTGCATAAAATTGCGATTGGCAAAACCATCTGATTTCAAAAAATCTAATATTGTTTTGCCAGATTGCTTATCCCTAATACCATTAATCAATTTTCGAGACAAACGTCCCCAACCAG

Synthesis fragment two

AATACCATTAATCAATTTTCGAGACAAACGTCCCCAACCAGTATAACGGCGACGTTTAAGCTGTTTCATCACCTTATCATCAAAGAGGTGAGCATATGTTTTAAGTCTTTCCTCAATCATCTCCCTATCTTCAAATAAGGTCAATGTTAAAACAATATCCTCTAAGATATCTTCATTTTCTTCATTATCCAAAAAATCTTTATCTTTAATAATTTTTAGCAAATCATGGTAGGTACCTAATGAAGCATTAAATCTATCTTCAACTCCTGAAATTTCAACACTATCAAAACATTCTATTTTTTTGAAATAATCTTCTTTTAATTGCTTAACGGTTACTTTTCGATTTGTTTTGAAGAGTAAATCAACAATGGCTTTCTTCTGTTCACCTGAAAGAAATGCTGGTTTTCGCATTCCTTCAGTAACATATTTGACCTTTGTCAATTCGTTATAAACCGTAAAATACTCATAAAGCAAACTATGTTTTGGTAGTACTTTTTCATTTGGAAGATTTTTATCAAAGTTTGTCATGCGTTCAATAAATGATTGAGCTGAAGCACCTTTATCGACAACTTCTTCAAAATTCCATGGGGTAATTGTTTCTTCAGACTTCCGAGTCATCCATGCAAAACGACTATTGCCACGCGCCAATGGACCAACATAATAAGGGATTCGAAAAGTCAAGATTTTTTCAATCTTCTCACGATTGTCTTTTAAAAATGGATAAAAGTCTTCTTGTCTTCTCAAAATAGCATGCAGCTCACCCAAGTGAATTTGATGGGGAATAGAGCCGTTGTCAAAGGTCCGTTGCTTGCGCAGCAAATCTTCACGATTTAGTTTCACCAATAATTCCTCAGTACCATCCATTTTTTCTAAAATTGGTTTGATAAATTTATAAAATTCTTCTTGGCTAGCTCCCCCATCAATATAACCTGCATATCCGTTTTTTGATTGATCAAAAAAGATTTCTTTATACTTTTCTGGAAGTTGTTGTCGAACTAAAGCTTTTAAAAGAGTCAAGTCTTGATGATGTTCATCGTAGCGTTTAATCATTGAAGCTGATAGGGGAGCCTTAGTTATTTCAGTATTTACTCTTAGGATATCTGAAAGTAAAATAGCATCTGATAAATTCTTAGCTGCCAAAAACAAATCAGCATATTGATCTCCAATTTGCGCCAATAAATTATCTAAATCATCATCGTAAGTATCTTTTGAAAGCTGTAATTTAGCATCTTCTGCCAAATCAAAATTTGATTTAAAATTAGGGGTCAAACCCAATGACAAAGCAATGAGATTCCCAAATAAGCCATTTTTCTTCTCACCGGGGAGCTGAGCAATGAGATTTTCTAATCGTCTTGATTTACTCAATCGTGCAGAAAGAATCGCTTTAGCATCTACTCCACTTGCGTTAATAGGGTTTTCTTCAAATAATTGATTGTAGGTTTGTACCAACTGGATAAATAGTTTGTCCACATCACTATTATCAGGATTTAAATCTCCCTCAATCAAAAAATGACCACGAAACTTAATCATATGCGCTAAGGCCAAATAGATTAAGCGCAAATCCGCTTTATCAGTAGAATCTACCAATTTTTTTCGCAGATGATAGATAGTTGGATATTTCTCATGATAAGCAACTTCATCTACTATATTTCCAAAAATAGGATGACGTTCATGCTTCTTGTCTTCTTCCACCAAAAAAGACTCTTCAAGTCGATGAAAGAAACTATCATCTACTTTCGCCATCTCATTTGAAAAAATCTCCTGTAGATAACAAATACGATTCTTCCGACGTGTATACCTTCTACGAGCTGTCCGTTTGAGACGAGTCGCTTCCGCTGTCTCTCCACTGTCAAATAAAAGAGCCCCTATAAGATTTTTTTTGATACTGTGGCGGTCTGTATTTCCCAGAACCTTGAACTTTTTAGACGGAACCTTATATTCATCAGTGATCACCGCCCATCCGACGCTATTTGTGCCGATAGCTAAGCCTATTGAGTATTTCTTATCCATGAAACGGCCTCCCAGATCTGTTAACGGTACCATCAAGCTTATTTTAATTATACTCTATCAATGATAGAGTGTCAATATTTTTTTTAGTTTTTCATGAACTCGAGGGGATCCAAATAAAAAACTAGTTTGACAAATAACTCTATCAATGATATAATGTCAACAAAAAGGAGGAATTAATGATGTCTAGATTAGATAAAAGTAAAGTGATTAACAGCGCATTAGAGCTGCTTAATGAGGTCGGAATCGAAGGTTTAACAACCCGTAAACTCGCCCAGAAGCTAGGTGTAGAGCAGCCTACATTGTATTGGCATGTAAAAAATAAGCGGGCTTTGCTCGACGCCTTAGCCATTGAGATGTTAGATAGGCACCATACTCACTTTTGCCCTTTAGAAGGGGAAAGCTGGCAAGATTTTTTACGTAATAACGCTAAAAGTTTTAGATGTGCTTTACTAAGTCATCGCGATGGAGCAAAAGTACATTTAGGTACACGGCCTACAGAAAAACAGTATGAAACTCTCGAAAATCAATTAGCCTTTTTATGCCAACAAGGTTTTTCACTAGAGAATGCATTATATGCACTCAGCGCTGTGGGGCATTTTACTTTAGGTTGCGTATTGGAAGATCAAGAGCATCAAGTCGCTAAAGAAGAAAGGGAAACACCTACTACTGATAGTATGCCGCCATTATTACGACAAGCTATCGAATTATTTGATCACCAAGGTGCAGAGCCAGCCTTCTTATTCGGCCTTGAATTGATCATATGCGGATTAGAAAAACAACTTAAATGTGAAAGTGGGTCTTAACTGCA
