# Supplementary material for: Copper ions inhibit pentose phosphate pathway function in Staphylococcus aureus
Source: PLoS Pathog. 2023 May 26;19(5):e1011393. doi: 10.1371/journal.ppat.1011393 (PMC10249872; doi:10.1371/journal.ppat.1011393)
Supplement: S5 Table — (DOCX) [file ppat.1011393.s016.docx]

| **S5 Table. primers used in this study** | | |
| --- | --- | --- |
| **primer name** | **DNA sequence** |  |
| upp KpnI fwd | tcggtaccttaggaggatgattatttATGAGTAAAGTACACGTTTTCGATCATC |  |
| upp BamHi rev | gaggatccTGTTCCAAAGCTTTAACTAAAGGAGCC |  |
| hpt KpnI fwd | tcggtaccttaggaggatgattatttATGAATAGCGTTATGCATAATGATTTG |  |
| hpt BamHi rev | ctagaggatccGTCTAAAATTAAATATATATGAAATACATTTTTCATAT |  |
| hisG BamHi rev | gaggatccTCCATGAACTTTAACTTCCTGACAAA |  |
| HisG KpnI fwd | ctcggtaccttaggaggatgattatttATGTTAAGAATCGCCATAGCCAAAG |  |
| purR veri fwd | GTGGTGCAGATGGTGCGTT |  |
| purR veri rev | TCTGTGCTTGAACATCAGCGC |  |
| purF KpnI fwd | tcggtaccttaggaggatgattatttATGTTTAACTACTCAGGATTAAACGAAGAA |  |
| purF BamHi rev | tctagaggatccACCGATAACTTCTTTACGCATCG |  |
| SAUSA300_1894 KpnI fwd | aactcggtaccttaggaggatgattatttGTGTACCAATTAGAAGACGACAG |  |
| SAUSA300_1894 BamHi rev | tctagaggatccTTCTTCAGCACTATCGATACGC |  |
| pyrE BamHi rev | actctagaggatccTTTGAATGATTGATAAATTCGTTACAATGTT |  |
| pLL39 *XbaI* 1902 fwd | AGCTCGGTACCCGGGTTTTGCAACAAAAATTTTGTGGAAGCATA |  |
| pLL39-1902 *Pst*I rev | CTGCAGGTCGACTCTAGAGGATCCCCTGAGACAAAAATAAACCATGCTTCCATAATTTG |  |
| pyrE KpnI fwd | gctcggtaccttaggaggatgattatttATGGCTAAAGAAATTGCAAAATCATTA |  |
| YCCaptfor | TATAGCGTAACTATAACGGTCCTAAtgtgcgctagcGGATGAAAGAATTATCTAAAACA |  |
| apttetRfor | GAGGAAATATTAATGGATTTAAAGCacgcgtCGGATTTTATGACCGATGATGAAGAAAA |  |
| apttetRrev | TTTTCTTCATCATCGGTCATAAAATCCGacgcgtGCTTTAAATCCATTAATATTTCCTC |  |
| tetaptRfor | TATATAAACATTCTCAAAGGGATTTCTAAacgcgtCCTTCATCAAATGTATAAGAACCA |  |
| tetaptRrev | TGGTTCTTATACATTTGATGAAGGacgcgtTTAGAAATCCCTTTGAGAATGTTTATATA |  |
| pJB38aptrev | actctagaggatccccgggtaccgagctcgaattcGCCGTATCTTCTAGTTCCCATTTG |  |
| 1591EcoRI | GGGGAATTCttaggaggatgattatttATGGATTTAAAGCAATACGTATCAGAAGTTC |  |
| 1591BamHI | CCCGGATCCCTTCGCCTTAAACAATGATTTAGGTACTC |  |
| Prs qpcr down fwd | GACCAAATGTTGCTGAAGTGATG |  |
| Prs qpcr down REV | GCTTGTGCAGCTAAAGTGATTG |  |
| Prs qPCR up fwd | GCATTAGCGCAAGAAGTTGCTGA |  |
| Prs qPCR up rev | CGTCACAACCACGAATACTCTCTTCGA |  |
| dCas qPCR fwd | CCCTATTAACGCAAGTGGAGTAG |  |
| dCas qPCR rev | GGGAGCTGAGCAATGAGATT |  |
| verify sgRNA fwd | CAGTGTGACTCTAGTAGAGAGCG |  |
| verify Cas fwd | GCAGGGCTACCAGCTAAATTTG |  |
| tetR verify fwd | GATCACCAAGGTGCAGAGC |  |
| PrsA12 sgRNA oligo1 | CTACTAATTTAGCAGTGATTGGCTCA |  |
| PrsA12 sgRNA oligo2 | AACTGAGCCAATCACTGCTAAATTAG |  |
| PrsA13 sgRNA oligo1 | CTAATGTTCATCACTTCAGCAACATT |  |
| PrsA13 sgRNA oligo2 | AAcAATGTTGCTGAAGTGATGAACAT |  |
| hla-sgRNA4-oligo1 | CTACTCAGTAACAACAACACTATTGCT |  |
| hla-sgRNA4-oligo2 | AACAGCAATAGTGTTGTTGTTACTGAG |  |
| pOS F2 fwd | TTGGGCGCTCTCGCTTCCTCGCTCACTG |  |
| pOS F2 rev | GCTTTTTTGAATTCAAGCTTATGGTGCACTCTC |  |
| pOS F1 rev | TCAGTGAGCGAGGAAGCGAGAGCGCCCAATACGC |  |
| pOS F1 fwd | AGTGGGTCTTAACTGCAGCGTTGCGCTCACT |  |
| pOS gibson rev2 | GTGAGCGCAACGCTGCAGTTAAGACCCACTTTCACA |  |
| pOS CrispR gibson fwd2 | AGTGCACCATAAGCTTGAATTCAAAAAAGCACCGACTC |  |
| mid rev 2 gibson | TTGTCTCGAAAATTGATTAATGGTATTAGGGATAAG |  |
| Crispr mid fwd | ATACCATTAATCAATTTTCGAGACAAACGTCCCCAACCAG |  |
| pOS CrispR gibson rev | AGCGAGGTGCAGTTAAGACCCACTTTCACATTTA |  |
| pOS verify fwd | TGCAGCTCCCGGAGAC |  |
| verify sgRNA fwd | CAGTGTGACTCTAGTAGAGAGCG |  |
| verify Cas fwd | GCAGGGCTACCAGCTAAATTTG |  |
| tetR verify fwd | GATCACCAAGGTGCAGAGC |  |
| verify dcas9 2 fwd | CTCCTTGGAGAATCCGCCTG |  |
| verify dcas9 3 fwd | CAGACTTCCGAGTCATCCATGC |  |
| verify dcas9 4 fwd | AAGCGCAAATCCGCTTTATCAG |  |
| Prs5BamHI | gggGGATCCATGTTAAATAATGAATATAAGAATTCGTCATT |  |
| Prs3SalI | gggGTCGACCGCCTTTTAAATATTAGTCAAATAATACGC |  |
